# The effectiveness of the Structured Health Intervention For Truckers (SHIFT): a cluster randomised controlled trial (RCT)

**DOI:** 10.1186/s12916-022-02372-7

**Published:** 2022-05-24

**Authors:** Stacy A. Clemes, Veronica Varela-Mato, Danielle H. Bodicoat, Cassandra L. Brookes, Yu-Ling Chen, Charlotte L. Edwardson, Laura J. Gray, Amber J. Guest, Vicki Johnson, Fehmidah Munir, Nicola J. Paine, Gerry Richardson, Katharina Ruettger, Mohsen Sayyah, Aron Sherry, Ana Suazo Di Paola, Jacqui Troughton, Thomas Yates, James A. King

**Affiliations:** 1 School of Sport, Exercise and Health Sciences, Loughborough University, Loughborough, LE11 3TU UK; 2 NIHR Leicester Biomedical Research Centre, Leicester, LE5 4PW UK; 3 Independent Researcher, Leicester, LE7 3SX UK; 4 Leicester Clinical Trials Unit, University of Leicester, Leicester, LE1 7RH UK; 5 Diabetes Research Centre, University of Leicester, Leicester, LE5 4PW UK; 6 Department of Health Sciences, University of Leicester, Leicester, LE1 7RH UK; 7 Leicester Diabetes Centre, University Hospitals of Leicester NHS Trust, Leicester, LE5 4PW UK; 8 Centre for Health Economics, University of York, York, YO10 5DD UK

**Keywords:** Physical activity, Sedentary behaviour, Occupational drivers, Diet, Obesity, Workplace

## Abstract

**Background:**

Long distance heavy goods vehicle (HGV) drivers exhibit higher than nationally representative rates of obesity, and obesity-related co-morbidities, and are underserved in terms of health promotion initiatives. The purpose of this study was to evaluate the effectiveness of the multicomponent ‘Structured Health Intervention For Truckers’ (SHIFT), compared to usual care, at 6- and 16–18-month follow-up.

**Methods:**

We conducted a two-arm cluster RCT in transport sites throughout the Midlands, UK. Outcome measures were assessed at baseline, at 6- and 16–18-month follow-up. Clusters were randomised (1:1) following baseline measurements to either the SHIFT arm or usual practice control arm. The 6-month SHIFT programme included a group-based interactive 6-h education and behaviour change session, health coach support and equipment provision (Fitbit® and resistance bands/balls to facilitate a ‘cab workout’). The primary outcome was device-assessed physical activity (mean steps/day) at 6 months. Secondary outcomes included the following: device-assessed sitting, physical activity intensity and sleep; cardiometabolic health, diet, mental wellbeing and work-related psychosocial variables. Data were analysed using mixed-effect linear regression models using a complete-case population.

**Results:**

Three hundred eighty-two HGV drivers (mean ± SD age: 48.4 ± 9.4 years, BMI: 30.4 ± 5.1 kg/m^2^, 99% male) were recruited across 25 clusters (sites) and randomised into either the SHIFT (12 clusters, *n* = 183) or control (13 clusters, *n* = 199) arms. At 6 months, 209 (55%) participants provided primary outcome data. Significant differences in mean daily steps were found between groups, in favour of the SHIFT arm (adjusted mean difference: 1008 steps/day, 95% CI: 145–1871, *p* = 0.022). Favourable differences were also seen in the SHIFT group, relative to the control group, in time spent sitting (− 24 mins/day, 95% CI: − 43 to − 6), and moderate-to-vigorous physical activity (6 mins/day, 95% CI: 0.3–11). Differences were not maintained at 16–18 months. No differences were observed between groups in the other secondary outcomes at either follow-up.

**Conclusions:**

The SHIFT programme led to a potentially clinically meaningful difference in daily steps, between trial arms, at 6 months. Whilst the longer-term impact is unclear, the programme offers potential to be incorporated into driver training courses to promote activity in this at-risk, underserved and hard-to-reach essential occupational group.

**Trial registration:**

ISRCTN10483894 (date registered: 01/03/2017)

**Supplementary Information:**

The online version contains supplementary material available at 10.1186/s12916-022-02372-7.

## Background

The UK Logistics sector contributes over £13 billion to the UK economy and employs approximately 300,000 heavy goods vehicle (HGV) drivers [1]. Due to the nature of their occupation, long-distance HGV drivers are exposed to a multitude of health-related risk factors and have been identified as working within one of the most hazardous professions in terms of health risks [2–4]. Their working environment and job demands present barriers to adopting a healthy lifestyle, leaving drivers vulnerable to a myriad of physical health conditions [5]. A systematic review has shown that HGV drivers globally exhibit high levels of physical inactivity, accumulate large volumes of sedentary (sitting) behaviour and are exposed to poor dietary choices [5]. Long, variable working hours, including shift work, contributes to sleep deprivation [6] and metabolic disturbances, further promoting uptake of unhealthy behavioural choices [3, 6]. This poor health profile is linked with high rates of obesity and cardiometabolic risk [5, 7, 8]. The incidence of obesity-related co-morbidities in HGV drivers is increasing, suggesting the trajectory of HGV driver health is declining [2, 3, 9–11]. Overall, this can culminate in HGV drivers having an increased risk of accidents, alongside higher rates of chronic diseases and reduced life expectancies compared to other occupational groups [2, 4, 12, 13].

To compound the high-risk health profile of HGV drivers [5, 7, 8], UK HGV drivers are an ageing workforce [14]. An All Party Parliamentary Group report highlighted the “demographic time bomb” the logistics industry is facing and the health impact of an ageing, at-risk, workforce “driving a vehicle often referred to as ‘a 40-tonne missile’” [15]. The UK Logistics sector is experiencing a serious short-fall in HGV drivers, described as reaching “a crisis point”, with the shortage rising from 60,000 in 2015 [16] to 100,000 in 2021 [17]. Reported barriers to driver recruitment include the lack of roadside facilities, medical concerns (e.g. perceived increased risk of obesity and diabetes due to the nature of the job), long and anti-social working hours [15]. There is an urgent need to address working conditions and the poor health profile of this ageing workforce to attract employees to the role. To date, HGV drivers have been underserved in terms of health promotion efforts [5, 18].

The lack of high-quality interventions in the HGV driver population [18] led us to develop the ‘Structured Health Intervention For Truckers’ (the SHIFT programme), a multicomponent, theory-driven, health behaviour intervention designed to promote physical activity and positive lifestyle changes in HGV drivers [19]. Initial piloting of our intervention, over a 3-month period, revealed increases in daily step counts (by an average of 1646 (SD 2156) steps/day) and fruit and vegetable intake, along with favourable changes in markers of cardiometabolic health [20]. The current study extends this work by evaluating the SHIFT programme within a cluster randomised controlled trial (RCT). The primary objective was to evaluate the effectiveness of the SHIFT programme, compared to usual care, on device-assessed physical activity (expressed as steps/day) at 6-month follow-up in a sample of HGV drivers. Secondary objectives were to explore the impact of the programme at 6- and 16–18-month follow-up on a range of activity and health-related secondary outcomes.

## Methods

### Study design

This study was a cluster RCT incorporating an internal pilot and included mixed-methods process and economic evaluations (which will be reported separately). Transport sites, located throughout the Midlands, UK, were the study setting. These consisted of independent companies whose logistics operations were subcontracted to an international logistics company. Individual transport depots were randomised to reduce contamination. The full trial protocol has been published [19]. The trial was approved by the Loughborough University Ethics Approvals (Human Participants) Sub-Committee (Reference: R17-P063). Loughborough University sponsored the study.

### Recruitment

Sites were eligible if they employed at least 20 long-distance HGV drivers (i.e. drivers who cover long distances with few delivery stops) and located within a 2-h drive of Loughborough University. Depots containing short-haul drivers who made frequent stops were excluded. Sites were identified by the company. Posters were displayed advertising the study, and drivers were provided with a participant information sheet for consideration. All drivers at each depot were eligible to participate (confirmed by completion of a health screening questionnaire in the presence of a researcher) irrespective of their current levels of physical activity, except for those with clinically diagnosed heart disease, haemophilia, blood-borne viruses or significant mobility limitation (which would prevent individuals from increasing physical activity levels). All participants provided written informed consent before undertaking any study procedures.

### Randomisation and masking

After baseline data collection, an independent statistician (blinded to cluster features) randomised depots (1:1). Randomisation was stratified by cluster size (< 40 vs. ≥ 40 drivers). Randomisation occurred in two phases, initially as part of the internal pilot, and secondly as part of the main trial. Given the nature of the intervention, participants were unable to be blinded to their group allocation. Participants and researchers were blinded to primary outcome data (device-measured physical activity), with participants’ group concealed during the processing of these data.

### Intervention

The Structured Health Intervention For Truckers (SHIFT) programme is a multi-component lifestyle-behaviour intervention designed to target behaviour changes in physical activity, diet and sitting in HGV drivers. Full details of the programme are described elsewhere [19]. In brief, the 6-month intervention, grounded within the Social Cognitive Theory for behaviour change [21], began with a group-based (4–6 participants) 6-h structured education session. The education session was delivered by two trained facilitators, utilising a written curriculum, and designed to support drivers to acquire knowledge about the links between physical activity (the primary focus), diet and sitting and type 2 diabetes and cardiovascular disease risk. The educational component is founded on the approach used in the award-winning suite of DESMOND programmes [22], used throughout the NHS, whilst being tailored for HGV drivers [23]. Within the education session, participants were supported to gain knowledge and develop skills relating to healthy lifestyle choices through group discussions and activities. For example, participants were encouraged to discuss feasible strategies to increase their physical activity, improve their diet and reduce their sitting time (when not driving) during working and non-working hours. Group-based activities were also undertaken to help drivers gain knowledge relating to the energy and sugar content of different foods and drinks. During the education session, participants were encouraged to develop individual goals and action plans, based on detailed individual feedback received during their baseline measurements, to achieve over the 6-month intervention. The education session was supported by specially developed resources and support materials.

During the education session, participants were provided with a Fitbit® Charge 2 (Fitbit, Inc., San Francisco, CA, USA) self-monitoring device to provide real-time feedback on activity levels. They were encouraged to use this to set goals (agreed at the session) to gradually increase their physical activity predominately through walking-based activity. Participants were provided with instructions on how to link their Fitbit account to an online monitoring system (Fitabase, Small Steps Labs LLC, San Diego, CA, USA). Participants’ data on the Fitabase website was only accessible to two members of the research team who used the step count data to provide participants with individually tailored step count challenges, at 6-weekly intervals, via a text messaging service (TextMagic, TextMagic Ltd. Cambridge, UK).

A ‘cab workout’ was introduced and practised at the education session, and participants were provided with resistance bands and balls and grip strength dynamometers to take away. Participants were encouraged to undertake the cab workout during breaks when not permitted to leave their vehicle. Participants were able to keep the intervention tools beyond the 6-month intervention; however, the step count challenges and supportive text messages (see below) sent by members of the research team ended after the 6-month intervention period. A Logic Model detailing the underlying theory behind the intervention components has been published elsewhere [19].

Trained facilitators delivering the structured education session were members of the research team in collaboration with personnel from the partnering logistics company. Individuals from the company who co-delivered the education sessions were predominantly HGV drivers who also acted as driver trainers within each depot. The ‘driver trainers’ were trained by specialist educators from the Leicester Diabetes Centre and mentored by trained members of the research team. The education sessions took place within the intervention depots. Personnel co-delivering the education sessions were also trained to act as a local champion providing ongoing health coach support (i.e. providing social support and encouragement to help drivers increase their steps and improve diet quality) to intervention participants during the first 6 months with additional text messaging support provided by the research team.

### Control arm

Depots assigned to the usual practice control arm were asked to continue with their usual care conditions. Participants received an educational leaflet at the outset detailing the importance of healthy lifestyle behaviours (i.e. undertaking regular physical activity, breaking up periods of prolonged sitting, and consuming a healthy diet) for the promotion of health and well-being. Control participants completed the same study measurements as those in the intervention worksites, at the same time points and received the same health feedback as intervention participants immediately following their measurements.

### Outcome measures

Baseline measurements took place prior to randomisation, with follow-up assessments originally intended to take place at 6 and 12 months. Due to government restrictions related to the COVID-19 pandemic, follow-up at 12 months were rescheduled and completed between 16 and 18 months after randomisation. Moreover, outcomes at this assessment were limited to the primary outcome (device-assessed physical activity) and self-reported data, due to restrictions imposed on face-to-face data collection. Commensurately, the independent Trial Steering Committee advised that the primary outcome assessment should be changed from 12- to 6-month follow-up. Baseline and follow-up assessments were conducted either at the beginning or end of drivers’ shifts, by trained researchers. Drivers were required to not consume any food or drink (other than water) for at least 4-h before assessments.

### Primary outcome measure

The primary outcome was physical activity, expressed as average steps per day, at 6 months, measured using the activPAL micro accelerometer (PAL Technologies Ltd, Glasgow, Scotland, UK), which provides a valid measure of walking and posture (i.e. sitting and standing) in adults [24]. Steps per day was chosen as the primary physical activity outcome variable because the intervention predominantly focused on the promotion of walking-based activity.

Devices were worn continuously (24 h/day) for 8 days. Devices were waterproofed and attached to the midline anterior aspect of participants’ non-dominant thigh using hyperfix transparent dressing (BSN Medical, Hull, UK). Participants were provided with a daily logbook and were required to record the times when they got into bed, went to sleep, woke-up and got out of bed. Participants also recorded whether each day was a workday or non-workday and whether the device had been removed at any times. On completion, devices and logbooks were returned to depots. Data were subsequently downloaded by a member of the research team and visually checked for adequate wear time. If an insufficient number of valid days were obtained, participants were contacted and asked to re-wear the device.

activPALs were initialised at 20 Hz and downloaded using manufacturer proprietary software (activPAL Professional v.7.2.38, PAL Technologies Ltd, Glasgow, UK). Event files were generated and processed using the freely available Processing PAL software (https://github.com/UOL-COLS/ProcessingPAL, version 1.3, University of Leicester, Leicester, UK) [25]. Once data were processed, heat maps were created showing valid waking wear data and invalid data and visually checked independently by two researchers. Suspected misclassifications were queried against participants self-reported logbook wake and sleep times. Where a misclassification was confirmed, data were corrected [26]. Summary variables were then calculated (listed under the “Secondary outcomes” section). A valid activPAL wear day was defined as having ≥ 10 h wear time per day, ≥ 1000 steps per day and < 95% of the day spent in any one behaviour (e.g. sitting, standing, or stepping). Participants were included in the primary outcome analysis if they provided at least one valid wear day at both baseline and 6 months. One valid day was chosen to maximise our sample and is in-line with previous studies [27, 28]

### Secondary outcomes

Secondary outcomes, described in detail elsewhere [19], included activPAL-assessed time spent sitting, standing, stepping, time in moderate-vigorous physical activity (MVPA) (stepping cadence ≥ 100 steps/min for ≥ 1 min) and time in light intensity physical activity (LPA) (calculated by subtracting sitting, standing and MVPA time from valid waking wear time). For each variable, daily averages were calculated across all valid days, as well as for workdays and non-workdays.

Sleep duration and efficiency were assessed using a GENEActiv tri-axial accelerometer (ActivInsights Ltd., Huntingdon, UK), worn (concurrently with the activPAL) on the non-dominant wrist continuously for 8 days. Sleep duration was calculated using a validated sleep detection algorithm [29]. A device wear time of ≥ 16 h per 24-h period was required to determine a valid night of sleep data, and participants were required to have provided at least one valid wear period at both baseline and 6 months to be included in the analyses. Sleep duration and efficiency data were summarised across all monitored days, for workdays and non-workdays. Full details of this measure are described elsewhere [30].

Height was measured, without shoes, using a portable stadiometer (Seca 206, Birmingham, UK). Body mass and body fat percentage were assessed via bio-electrical impedance analysis using portable scales (DC-360S, Tanita Corporation, Tokyo, Japan). Waist, hip, and neck circumferences were measured using standard anthropometric measuring tape (Seca, Birmingham, UK), and waist-to-hip ratio was calculated.

Capillary blood samples were collected via finger-prick blood sampling and analysed using validated point-of-care analysers for glycated haemoglobin (HbA1c) (A1CNow®+, PTS Diagnostics, Indianapolis, USA), triacylglycerol, total cholesterol and HDL-cholesterol (Cardiocheck®, PTS Diagnostics, Indianapolis, USA). LDL-cholesterol was calculated using the Friedewald formula [31]. Resting blood pressure and heart rate was measured using an automated sphygmomanometer (HEM-907, Omron Corporation, Japan); three measurements were taken at 5-min intervals and mean values calculated from the second and third readings. Blood pressure and heart rate were also measured under a simulated stress task, involving completion of the Stroop Test (also providing a measure of cognitive function [32]) and the Mirror Tracing task [33]. This was designed to provide a measure of psychophysiological reactivity, described elsewhere [34]. Grip strength (kg) was assessed from both hands using the Takei Hand-Grip dynamometer (Takei Scientific Instruments Co., Ltd; Japan).

At each assessment, participants completed a questionnaire booklet where they provided self-reported information on dietary intake, musculoskeletal symptoms, mental wellbeing, work-related psychosocial variables, medication use, smoking status and alcohol intake [19]. Demographic information, including date-of-birth, sex, ethnicity, highest level of education, marital status, medication use, postcode (to determine Index of Multiple Deprivation [IMD] as an indicator of neighbourhood socioeconomic status), working hours, years worked as an HGV driver, shift pattern and years worked at the logistics company was also collected at baseline [19]. Participants and transport managers were asked to report any adverse events or serious adverse events to the research team, either via the trial’s text messaging service, or via telephone (using a phone number provided).

### Sample size

Our earlier exploratory pre-post study revealed that, on average, HGV drivers accumulated 8786 steps per day across both workdays and non-workdays with a standard deviation of 2919 steps [20]. This trial was powered to look for a difference in step counts (the primary outcome) of 1500 steps per day (equivalent to approximately 15 min of moderately paced walking) between the intervention and control group, given evidence demonstrating a linear association between step counts and a range of morbidity-related outcomes [35–37].

Based on a cluster size of 10, a conservative intra-class correlation coefficient (ICC) of 0.05 (as there were no previous data to inform this, we were guided by recommendations of Campbell et al. [38]), an alpha of 0.05, power of 80% and a coefficient of variation to allow for variation in cluster size of 0.51 (based on information provided by the company), we required 110 participants from 11 clusters per arm. The sample size was inflated by 30% to allow for drop-out. The number of clusters was also inflated by 2 to allow for whole cluster drop-out. We therefore aimed to recruit 24 clusters (transport sites) with an average of 14 participants per cluster, providing a total target sample size of 336 drivers. Due to one pilot site not allowing participants to wear accelerometers during working hours for health and safety reasons, thus limiting the collection of the primary outcome measure (activPAL-determined steps/day) to non-working hours only, the Trial Steering Committee approved the recruitment of an additional site in the main trial phase (in November 2018).

### Statistical analysis

A detailed statistical analysis plan was agreed before the data were released. Due to the number of clusters [39], the primary analysis was performed using a mixed effects linear regression model with each participant’s daily average number of steps (measured using the activPAL, across any valid day(s)) at 6 months as the outcome, adjusting for their daily average number of steps at baseline and for the average waking wear time at baseline and 6 months. The model also included a categorical variable for randomisation group (control as reference) and a term for the stratification factor (cluster size; small < 40 or large ≥ 40). Depot was included as a random effect. The primary analysis examined the effect of the intervention using a complete case population. All clusters randomised and the recruited participants in these clusters, excluding those with missing outcome data (i.e. without at least 1 valid day of activPAL data at baseline and follow-up), were included in the primary analysis with participants analysed in the arm to which they were randomised. The estimate of the adjusted difference between the SHIFT arm and the control arm for daily average number of steps at 6 months and the corresponding 95% confidence intervals and *p*-values were presented. The cluster ICC was estimated to assess the strength of the clustering effect.

Sensitivity analyses were performed to assess the impact of missing data on the primary results and to account for uncertainty associated with imputing data (full intention-to-treat (ITT) analysis). Missing data from variables included in the primary analysis model (average daily steps at baseline and 6 months) were imputed using multiple imputation. The following variables were included in the imputation model: baseline BMI, sex, ethnicity, age, cluster size category, years worked as HGV driver and average waking wear time across baseline and 6 months. Missing values for these predictor variables were also imputed if needed. Twenty imputations were made and combined using Rubin’s rules [40]. Additional worst- and best-case scenario ITT analyses using basic imputation methods were also carried out. In the worst-case scenario, missing outcome data in the final analysis model (at baseline and 6 months) were replaced using the mean for the usual care arm, whilst in the best-case scenario data were replaced using the mean from the respective arm. We carried out further sensitivity analyses by assessing the effect of the number of valid activPAL days on the primary outcome analysis. This analysis was performed by including participants who provided valid activPAL data on at least 2, 3 and 4 valid days at baseline and 6 months.

Secondary outcomes were analysed using similar methodology to the primary outcome. Due to the volume of secondary outcomes assessed, formal statistical testing of secondary outcome variables was restricted to the following key secondary outcomes: steps/day (16–18 months follow-up), activPAL-determined time spent sitting, standing and stepping, and time in LPA and MVPA daily, across workdays and non-workdays (at 6- and 16–18 months). The models for each of these secondary outcomes were adjusted for their respective variable at baseline and for the respective average wear time period (i.e. daily, workdays or non-workdays) at baseline and follow-up.

Fruit and vegetable intake (grams/day) and dietary quality score were also analysed at 6- and 16–18 months. Furthermore, the following markers of cardiometabolic health were compared statistically at 6 months: weight, BMI, % body fat, waist circumference, glycated haemoglobin (mmol/mol), triglycerides (mmol/l), HDL cholesterol (mmol/l), LDL cholesterol (mmol/l) and total cholesterol (mmol/l). All models included a categorical variable for intervention group (control as reference) and the stratification factor (cluster size). No corrections for multiple testing were made. In all models, estimates of the difference between the SHIFT arm and the control arm for the variables examined are presented, along with corresponding 95% confidence intervals and *p*-values.

For the other secondary outcomes, continuous data that were approximately normally distributed were summarised in terms of the mean and standard deviation. Skewed data are presented in terms of the medians and inter-quartile range (IQR). Ordinal and categorical data are summarised in terms of frequency counts and percentages. All analyses were conducted using Stata version 16 (StataCorp LP, College Station, TX, USA). Statistical significance was set at 5%. All reported statistical tests are two sided.

## Results

Recruitment for the internal pilot began in August 2017 with baseline data collection occurring between January and August 2018 and follow-up data collection occurring between September 2018 and November 2019. Recruitment for the main trial phase began in January 2019 with baseline data collection occurring between February and July 2019 and follow-up data collection occurring between September 2019 and December 2020. The progression criteria for the internal pilot are detailed in Additional file 1: Table S1. Pilot trial outcomes relating to these criteria were reviewed by the Trial Steering Committee in December 2018, and the trial approved for continuation.

**Fig. 1:**
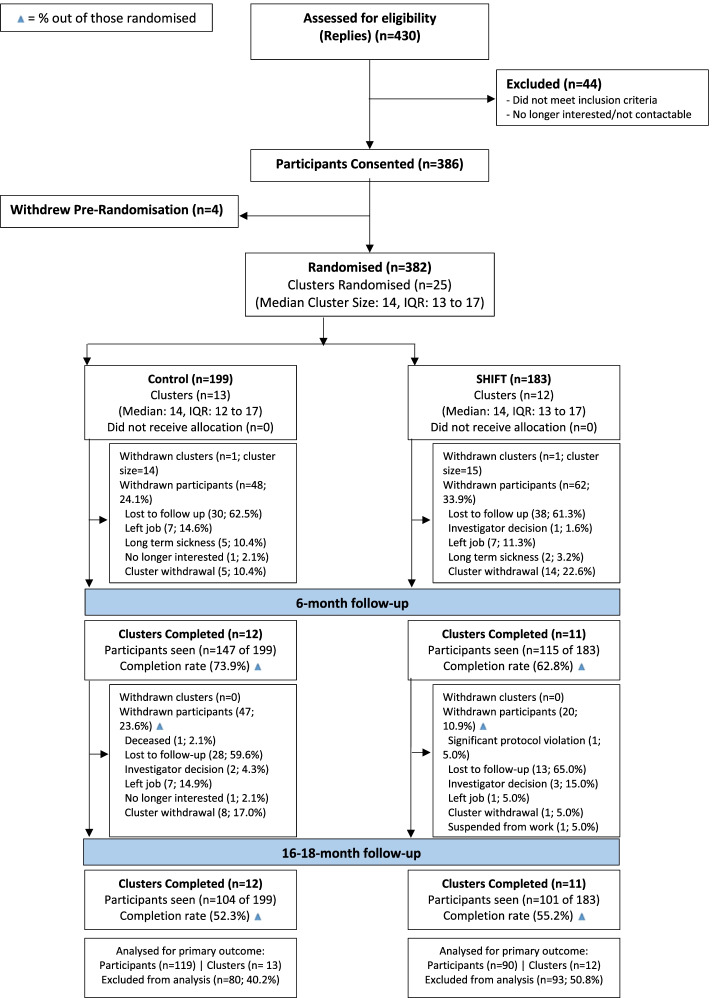
CONSORT diagram of participant flow through the study (IQR, inter-quartile range)

Figure 1 shows the flow of participants through the study, combining data from the internal pilot and main trial phases. Overall, 386 participants across 25 clusters were recruited and consented into the study. The 25 sites (1502 drivers were employed across these sites) operated within the transport, retail, hospitality, healthcare, pharmaceutical, construction, oil and gas and automotive industries. The included sites were a similar size and had a similar variation in size, to the company’s national-level data. There were 13 sites (clusters) randomised to the control arm (199 participants) and 12 sites (clusters) randomised to the SHIFT arm (183 participants); 4 participants withdrew prior to cluster randomisation. Median cluster size was 14 with an inter-quartile range (IQR) of 13 to 17. Between baseline and 6-month follow-up, 2 sites dropped out of the trial (1 intervention, 1 control) due to site closures. 31.4% and 46.3% of participants did not attend the 6-month and 16–18-month follow-up assessments, respectively. Within the intervention sites, 79.2% (*n* = 145) of participants attended the structured education session.

Characteristics of clusters and participants within each trial arm, and overall, are shown in Table 1. Descriptive comparisons between baseline characteristics of participants with complete primary outcome data (*n* = 209; 54.7%) are shown in Additional file 1: Table S2. There were no noticeable differences between completers (i.e. participants who provided valid activPAL data at baseline and 6 months) and non-completers in terms of cluster size, age, BMI, number of years as a HGV driver and number of steps/day at baseline.

**Table 1:** Cluster and participant characteristics per trial arm and overall, at baseline. Data are summarised as the median and inter-quartile range (IQR), unless otherwise stated

	Control Clusters = 13 Participants = 199	SHIFT Clusters = 12 Participants = 183	Overall Clusters = 25 Participants = 382	Missing values
**Cluster level**				
Cluster size category, *n* (%)				0
Small (< 40 drivers)	81 (40.7%)	93 (50.8%)	174 (45.6%)	
Large (≥ 40 drivers)	118 (59.3%)	90 (49.2%)	208 (54.4%)	
**Participant level**				
***Cluster size***				0
Median (IQR)	14 (12, 17)	14 (13, 17)	14 (13, 17)	
Min, max	9, 24	11, 25	9, 25	
***Age,*** Mean (SD)	48.3 (9.7)	48.6 (9.1)	48.4 (9.4)	2
***Sex***, *n* (%) male	196 (98.5%)	182 (99.5%)	378 (99.0%)	0
***Ethnicity***, *n* (%)				1
White British	154 (77.4%)	152 (83.1%)	306 (80.1%)	
Other ethnicity	45 (22.6%)	30 (16.4%)	75 (19.6%)	
***Shift pattern***^***a***^, *n* (%)				0
Morning	146 (73.4%)	124 (67.8%)	270 (70.7%)	
Afternoon	29 (14.6%)	31 (16.9%)	60 (15.7%)	
Night	35 (17.6%)	45 (24.6%)	80 (20.9%)	
***Duration working at company (years)***	6.17 (3.67, 11.50)	9.30 (4.06, 14.27)	7.75 (3.88, 13.42)	0
***Duration working as a HGV driver (years)***	15.00 (6.00, 26.00)	17.00 (10.00, 25.02)	16.00 (9.00, 25.17)	0
***Average hours worked per week***	48 (45, 50)	48 (45, 50)	48 (45, 50)	0
***IMD rank***	16779 (8500, 22904)	16040 (7934, 22171)	16591 (8165, 22544)	30
***Marital status***, *n* (%)				0
Married	133 (66.8%)	113 (61.8%)	246 (64.4%)	
Living with partner	34 (17.1%)	31 (16.9%)	65 (17.0%)	
Other (separated/divorced, single, widowed)	32 (16.1%)	39 (21.4%)	71 (18.6%)	
***Level of education***, *n* (%), degree or above	16 (8.0%)	10 (5.5%)	26 (6.8%)	8
***Diabetes history****, n* (%), yes^b^	15 (7.5%)	9 (4.9%)	24 (6.3%)	1
***Smoking status***, *n* (%)				1
Never smoked	73 (36.7%)	77 (42.1%)	150 (39.3%)	
Ex-smoker	84 (42.2%)	73 (39.9%)	157 (41.1%)	
Current smoker	42 (21.1%)	32 (17.5%)	74 (19.4%)	
***Anthropometric measures and markers of adiposity***				
Weight (kg)	94.0 (84.2, 106.9)	95.7 (84.0, 106.4)	94.8 (84.1, 106.5)	2
Body fat (%), mean (SD)	26.8 (5.8)	27.3 (6.0)	27.0 (5.9)	11
Fat mass (kg)	25.3 (19.6, 32.3)	25.6 (19.9, 32.7)	25.5 (19.6, 32.4)	11
Fat Free mass (kg), mean (SD)	69.3 (8.6)	69.6 (7.8)	69.5 (8.2)	12
Body mass index (kg/m^2^)	29.6 (27.0, 32.8)	29.9 (26.9, 33.7)	29.8 (26.9, 33.2)	2
Waist circumference (cm)	104.4 (94.6, 113.1)	103.0 (95.0, 113.5)	103.7 (95.0, 113.4)	2
Hip circumference (cm)	106.5 (101.0, 111.8)	107.5 (103.0, 114.0)	107.0 (102.0, 112.5)	2
Waist-hip ratio (cm), mean (SD)	0.97 (0.07)	0.97 (0.07)	0.97 (0.07)	2
Neck circumference (cm)	40.2 (38.9, 42.5)	41.0 (38.3, 42.5)	40.5 (38.4, 42.5)	2
***Resting blood pressure (BP) and heart rate***				
Systolic BP (mmHg)	130 (122, 140)	130 (122, 138)	130 (122, 139)	2
Diastolic BP (mmHg)	82 (76, 90)	81 (76, 88)	82 (76, 88)	2
Heart rate (bpm), mean (SD)	68 (10)	68 (10)	68 (10)	4
***Biochemical assessments***				
Glycated haemoglobin (mmol/mol)	35 (32, 38)	34 (31, 38)	35 (31, 38)	14
Glycated haemoglobin (%)	5.4 (5.1, 5.6)	5.3 (5.0, 5.6)	5.4 (5.0, 5.6)	14
Triglycerides (mmol/l)	1.3 (1.0, 2.1)	1.3 (0.9, 2.1)	1.3 (0.9, 2.1)	5
HDL cholesterol (mmol/l)	1.1 (1.0, 1.4)	1.1 (1.0, 1.4)	1.1 (1.0, 1.4)	5
LDL cholesterol (mmol/l)	2.8 (2.3, 3.5)	2.8 (2.4, 3.5)	2.8 (2.3, 3.5)	6
Total cholesterol (mmol/l)	4.4 (3.8, 5.1)	4.4 (3.8, 5.1)	4.4 (3.8, 5.1)	5
***Physical activity and sitting time***				41
Steps/day	8471 (6774, 10160)	8725 (7033, 11298)	8583 (6922, 10696)	
Sitting (mins/day), mean (SD)	678 (91)	651 (97)	665 (95)	
Standing (mins/day)	195 (165, 238)	213 (180, 244)	203 (169, 243)	
Stepping (mins/day)	112 (90, 134)	116 (93, 149)	114 (92, 139)	
MVPA (mins/day)	10 (6, 18)	11 (6, 21)	10 (6, 19)	
LPA (mins/day)	97 (81, 114)	102 (83, 129)	99 (82, 123)	
Number of valid days	8 (6, 8)	7 (5, 8)	7 (6, 8)	
Waking wear time (mins/day)	993 (955, 1033)	989 (950, 1022)	990 (953, 1032)	
***Sleep***				36
Sleep window duration (mins/day)	426 (393, 465)	424 (387, 459)	425 (390, 460)	
Sleep duration (mins/day)	371 (336, 405)	371 (340, 407)	371 (337, 406)	
Sleep efficiency (%)	88.5 (84.2, 91.3)	88.9 (84.6, 92.0)	88.6 (84.3, 91.5)	
Number of valid nights	6 (6, 6)	6 (5, 6)	6 (6, 6)	
***Fruit and vegetable intake and dietary quality***				111
Fruit intake (grams/day), mean (SD)	101 (122)	135 (158)	117 (141)	
Vegetable intake (grams/day), Mean (SD)	110 (135)	128 (166)	118 (150)	
Dietary quality score, mean (SD)	11.1 (2.0)	11.1 (2.1)	11.1 (2.0)	

^a^Approximately 90% of the sample were shift workers, where working hours did not fit entirely within the conventional 8 am–6 pm window. The categories applied (morning, afternoon, night) refer to the predominant period of the day (or night) that participants spend working

^b^In the control group, 14 of the 15 have type 2 diabetes; 1 did not report type. Eleven of the 15 controlled their diabetes with medical treatment, 3 with lifestyle only and 1 did not report control type. In the SHIFT group, all 9 had type 2 diabetes, 7 controlled their diabetes with medical treatment, 2 with diet only

### Primary outcome

Table 2 shows mean daily step counts measured across any valid day(s) at baseline and at 6 months, along with the changes in daily steps for the SHIFT and control groups. The primary complete-case analysis revealed a statistically significant difference in mean daily step counts at 6 months, in favour of the SHIFT group (adjusted mean difference: 1008 steps/day; 95% confidence interval (CI): 145 to 1871, *p* = 0.022). The ICC for the model was 0.112. No significant effects were seen in the ITT analyses, with the exception of the best-case scenario. Sensitivity analyses showed similar results to the primary analysis, with significant differences observed between groups in terms of daily step counts at 6 months, when including participants with ≥ 2, 3 and 4 valid days of activPAL data (Table 2).

**Table 2:** Changes in mean daily steps per day across any valid day/s at 6-month follow-up

	Number of clusters		Number of participants		Baseline Mean (SD)		6-month follow-up Mean (SD)		Mean change from baseline to 6 months (SD)		SHIFT vs. control at 6 months	
	Control	SHIFT	Control	SHIFT	Control	SHIFT	Control	SHIFT	Control	SHIFT	Adjusted mean difference (95% CI)^**a**^	***p***-value
Primary analysis (complete case)^b^	13	12	119	90	8932 (2922)	9355 (3305)	8216 (2767)	9387 (3455)	− 716 (2109)	32 (2939)	1008 (145, 1871)	0.022
*Intention to treat*^*b*^												
Multiple imputation ^c^	13	12	199	183	–	–	–	–	–	–	335 (− 471, 1141)	0.414
Worst case scenario ^d^	13	12	199	183	8788 (2843)	9394 (3134)	8244 (2270)	8846 (2527)	− 544 (2499)	− 548 (3056)	399 (− 129, 927)	0.139
Best case scenario ^e^	13	12	199	183	8788 (2843)	9464 (3127)	8244 (2270)	9344 (2466)	− 543 (2499)	− 120 (2985)	868 (398, 1338)	< 0.001
*Sensitivity analyses—effect of number of valid activPAL days (complete-case):*												
≥ 2 days	13	12	118	88	8960 (2919)	9427 (3291)	8238 (2768)	9411 (3488)	− 722 (2117)	− 16 (2949)	981 (102, 1860)	0.029
≥ 3 days	13	12	116	87	8925 (2921)	9459 (3296)	8278 (2770)	9456 (3484)	− 647 (2015)	− 4 (2963)	906 (27, 1784)	0.043
≥ 4 days	13	12	113	79	8832 (2877)	9385 (3290)	8179 (2710)	9480.69 (3509)	− 653 (2020)	96 (3028)	973 (76, 1870)	0.034

^**a**^Adjusted for steps per day at baseline, average waking wear time across baseline and 6 months, and cluster size category (small < 40; large ≥ 40) with a random effect for cluster (depot).

^**b**^≥ 1 valid day at baseline and 6 months

^**c**^Means (SDs) cannot be calculated for intention to treat population because multiple imputation methodology was used

^d^Missing outcome data in the final analysis model (at baseline and 6 months) was replaced using the mean for the standard care arm

^e^Missing outcome data in the final analysis model (at baseline and 6 months) was replaced using the mean from the respective arm

### Secondary outcomes

At 6 months, across any valid day(s), we also observed that sitting time was lower in the SHIFT arm compared to the control arm, whilst times spent standing, stepping and time in MVPA were higher (Table 3). There were no differences between groups in activPAL variables measured at 16–18 months (Table 3). There were no differences observed between groups in any activPAL variables measured across workdays at 6 or 16–18 months (Additional file 1: Table S3). At 6 months across non-workdays, differences in favour of the SHIFT arm were observed between groups in daily step counts, sitting time, time spent stepping, time in LPA and MVPA. There were no differences observed between groups in activPAL variables measured at 16–18 months across non-workdays (Additional file 1: Table S4).

**Table 3:** Changes in key daily activPAL variables across any valid day/s at 6- and 16–18-month follow-up

Daily variables (min/day)	Number of clusters		Number of participants		Baseline Mean (SD)		Follow-up Mean (SD)		Mean change from baseline to follow-up (SD)		SHIFT vs. control	
	Control	SHIFT	Control	SHIFT	Control	SHIFT	Control	SHIFT	Control	SHIFT	Adjusted mean difference (95% CI) ^**a**^	***p***-value
Steps per day ^b^												
16–18 months	12	10	90	74	8978 (3226)	9663 (3122)	8789 (3148)	9259 (3105)	− 189 (2169)	− 404 (2688)	94 (− 878, 1066)	0.849
Time sitting ^b^												
6 months	13	12	119	90	675 (92)	664 (92)	696 (87)	655 (93)	21 (79)	− 9 (77)	− 24 (− 43, − 6)	0.011
16–18 months	12	10	90	74	676 (97)	651 (87)	679 (98)	647 (77)	4 (82)	− 4 (90)	− 12 (− 34, 9)	0.268
Time standing ^b^												
6 months	13	12	119	90	204 (55)	209 (53)	194 (56)	210 (61)	− 10 (37)	1 (48)	14 (2, 26)	0.024
16–18 months	12	10	90	74	200 (54)	216 (61)	197 (58)	211 (64)	− 3 (45)	− 5 (77)	11 (− 5, 27)	0.183
Time stepping ^b^												
6 months	13	12	119	90	116 (34)	122 (40)	107 (32)	122 (40)	− 8 (23)	− 0.4 (32)	11 (1, 21)	0.024
16–18 months	12	10	90	74	117 (36)	125 (38)	114 (37)	120 (36)	− 2 (22)	− 5 (31)	1 (− 9, 11)	0.818
Time LPA ^b^												
6 months	13	12	119	90	101 (29)	107 (34)	94 (28)	104 (33)	− 6 (19)	− 3 (25)	5 (− 2, 12)	0.152
16–18 months	12	10	90	74	102 (29)	109 (34)	100 (32)	104 (32)	− 2 (18)	− 5 (26)	− 1 (− 8, 7)	0.863
Time MVPA ^b^												
6 months	13	12	119	90	15 (15)	15 (14)	13 (10)	18 (18)	− 2 (14)	3 (19)	6 (0.3, 11)	0.038
16–18 months	12	10	90	74	14 (15)	16 (14)	14 (13)	16 (16)	− 1 (15)	− 0.1 (16)	2 (− 3, 7)	0.539

^a^Adjusted for variable at baseline, average waking wear time across baseline and 6 (or 12) months, and cluster size category (small < 40; large ≥40) with a random effect for cluster (depot)

^b^≥ 1 valid day at baseline and 6 (or 16–18) months

There were no differences observed between groups in anthropometric measures, markers of cardiometabolic health or psychophysiological reactivity at 6 months (Table 4 and Additional file 1: Table S5). No differences between groups in reported fruit and vegetable intake or overall dietary quality were seen at 6 and 16–18 months (Table 4). No noticeable changes in grip strength were observed between baseline and 6 months in the control group, whereas modest improvements in grip strength for both hands were observed at 6 months in the SHIFT arm (Additional file 1: Table S6). Both groups exhibited a decrease in sleep duration between baseline and 6 months on workdays, whilst sleep duration increased for both groups at 6 months on non-workdays. There were no noticeable between group differences in changes in sleep duration or efficiency at 6 months (Additional file 1: Table S7). No noticeable between group differences in changes in cognitive function were observed at 6 months (Additional file 1: Table S8). There was a tendency for the prevalence of musculoskeletal discomfort across the majority of body sites to decrease at 6 and 16–18 months in both groups, with similar changes in prevalence and overall discomfort scores occurring between groups (Additional file 1: Table S9). Similarly, there were no noticeable between group differences in changes in reported mental wellbeing or work-related psychosocial variables at either follow-up (Additional file 1: Tables S10-12). No serious adverse events were reported throughout the trial.

**Table 4:** Changes in adiposity, biochemical, and dietary-related secondary outcomes at 6- and 16–18-month (dietary variables only) follow-up

Anthropometric measures	Number of clusters		Number of participants		Baseline Mean (SD)		Follow-up Mean (SD)		Mean change from baseline to follow-up (SD)^a^		SHIFT vs. control	
	Control	SHIFT	Control	SHIFT	Control	SHIFT	Control	SHIFT	Control	SHIFT	Adjusted mean difference (95% CI)^**a**^	***p***-value
Weight, kg												
6 months	13	12	143	112	94.9 (17.5)	96.9 (16.0)	94.8 (17.4)	95.5 (16.2)	− 0.1 (4.48)	− 1.4 (5.2)	− 1.2 (− 2.6, 0.1)	0.078
BMI, kg/m^2^												
6 months	13	12	143	112	29.9 (5.2)	30.7 (5.0)	29.9 (5.1)	30.3 (5.1)	− 0.0 (1.4)	− 0.4 (1.6)	− 0.4 (− 0.8, 0.1)	0.086
% Body fat												
6 months	13	10	141	96	26.3 (5.9)	27.3 (5.9)	26.4 (6.0)	27.1 (5.8)	0.1 (1.7)	− 0.2 (2.0)	− 0.2 (− 0.7, 0.3)	0.435
Waist circumference, cm												
6 months	13	11	143	103	103.7 (13.7)	104.9 (12.7)	103.8 (13.9)	103.6 (12.7)	0.1 (5.1)	− 1.3 (6.7)	− 1.1 (− 2.7, 0.5)	0.195
HbA1c, mmol/mol												
6 months	13	10	139	89	36.8 (9.4)	35.6 (10.3)	37.1 (10.4)	35.0 (9.0)	0.2 (6.0)	− 0.6 (6.9)	− 1.9 (− 4.9, 1.2)	0.229
Triglycerides, mmol/l												
6 months	13	10	143	98	1.7 (1.1)	1.6 (0.9)	1.7 (1.1)	1.7 (1.1)	0.1 (1.0)	0.04 (0.9)	− 0.08 (− 0.3, 0.2)	0.530
HDL cholesterol, mmol/l												
6 months	13	10	143	98	1.2 (0.4)	1.2 (0.3)	1.3 (0.3)	1.3 (0.3)	0.02 (0.2)	0.1 (0.2)	0.04 (− 0.02, 0.1)	0.241
LDL cholesterol, mmol/l												
6 months	13	10	143	98	2.9 (0.8)	2.8 (0.8)	2.9 (0.9)	2.8 (0.9)	− 0.01 (0.9)	− 0.03 (0.8)	0.0 (− 0.2, 0.2)	0.973
Total cholesterol, mmol/l												
6 months	13	10	143	98	4.4 (0.9)	4.4 (0.9)	4.5 (1.0)	4.4 (1.0)	0.02 (0.9)	0.1 (0.9)	0.02 (− 0.2, 0.2)	0.868
Fruit (grams/day)												
6 months	13	12	147	124	100.8 (122.2)	135.4 (158.1)	123.9 (142.3)	120.6 (153.6)	23.1 (128.1)	− 14.8 (159.5)	− 20.6 (− 64.5, 23.3)	0.359
16–18 months	12	10	112	102	92.2 (109.4)	127.4 (152.7)	89.6 (111.8)	112.1 (148.0)	− 2.7 (105.3)	− 15.2 (162.8)	7.4 (− 30.3, 45.2)	0.700
Vegetables (grams/day)												
6 months	13	12	147	124	110.5 (135.3)	127.7 (165.8)	106.1 (142.1)	131.9 (184.6)	− 4.4 (167.3)	4.1 (207.0)	27.3 (− 24.8, 79.4)	0.305
16–18 months	12	10	112	102	95.1 (98.1)	127.3 (167.8)	100.2 (145)	90.0 (101.0)	5.1 (148.5)	− 37.2 (157.4)	− 25.3 (− 68.5, 17.9)	0.251
Dietary quality score ^b^												
6 months	13	12	147	124	11.1 (2.0)	11.1 (2.1)	11.4 (1.7)	11.1 (2.0)	0.3 (2.2)	− 0.01 (2.4)	− 0.2 (− 0.7, 0.2)	0.241
16–18 months	12	11	112	102	11.1 (1.8)	11.0 (2.0)	11.3 (1.6)	11.3 (1.8)	0.1 (2.0)	0.3 (2.3)	0.07 (− 0.4, 0.5)	0.778

^a^Adjusted for variable at baseline

^b^Dietary quality score ranges from 5–15, with higher scores indicating higher dietary quality based on consumption of fruit, vegetables, oily fish, fats and non-milk extrinsic sugars

## Discussion

This trial evaluated the effectiveness of the multicomponent SHIFT programme in a sample of long-distance HGV drivers. Our primary analysis revealed that the SHIFT group accumulated 1008 more steps/day relative to the control group at 6 months. The programme also led to differences at 6 months between groups, in favour of the SHIFT arm, in drivers’ time spent sitting, standing, stepping and time in MVPA, with these differences particularly pronounced on non-workdays. However, differences between trial arms were not maintained at 16–18 months.

Whilst the difference in the primary outcome measure between the SHIFT and control arms at 6 months (1008 steps/day) was lower than 1500 steps/day which formed the basis of our sample size calculation, it has recently been reported that 500 steps/day is the minimum clinically important difference for inactive individuals, applying equally to men and women [41]. Therefore, the difference observed in the intervention group relative to the control group is potentially clinically meaningful and of a sufficient magnitude to impact longer-term health and mortality risk [41]. Whilst differences observed between groups at 6 months were largely driven by maintenance of baseline physical activity levels in the SHIFT arm and a decline in physical activity in the control arm, these differences remain potentially clinically important given physical inactivity is widely associated with an increased risk of many adverse health conditions [42]. Whilst reasons for the decline in physical activity levels observed in the control arm are unclear, preventing such a decline in habitual activity in any population/individual is important when considering longer-term health outcomes.

Although the significant differences between groups in activity levels did not persist into the longer-term, it is hard to draw conclusions given that the measurements were taken in the middle of a pandemic that had an impact on people’s working practices and behaviour. Aside from the pandemic, the lack of differences between groups at 16–18 months follow-up is consistent with that seen in other physical activity interventions with longer-term follow-up measures (> 12 months) [43]. Within HGV drivers, environmental, organisational and policy-level changes (e.g. provision of secure rest stops which facilitate engagement in physical activity, modifications of driving hour regulations) will likely be required in addition to programmes such as SHIFT to promote longer-term behavioural changes. Indeed, the absence of any significant differences in physical activity and sedentary behaviour on workdays between trial arms at 6 months suggests that due to the constraints of their job, participants in the SHIFT arm were more likely to adopt positive activity-related behaviours on non-workdays. For example, on non-workdays, relative to controls at 6 months, participants in the SHIFT arm accumulated 2012 more steps/day, an additional 10 min/day of light physical activity and 11 min/day of MVPA and 40 min/day less sitting.

Despite the high-risk health profile of HGV drivers globally [5], limited health promotion interventions have been conducted in this occupational group. A systematic review of health promotion interventions in HGV drivers (including only 8 studies) observed that interventions generally led to improvements in health and health behaviours; however, the review cautioned that the strength of the evidence was limited due to poor study designs, with no control groups, small samples and no or limited follow-up periods [18]. The present study addresses these limitations, being the first to formally evaluate a health promotion intervention within this at-risk occupational group, employing a cluster RCT design with an extended follow-up period.

Of the limited available literature, only one other study within HGV drivers has examined the potential impact of a wrist-worn device to help monitor and self-regulate physical activity levels and dietary choices [44]. In a sample of 26 Australian HGV drivers, Gilson et al. [44] observed that participants’ daily step counts remained constant across the 20-week intervention, averaging between 8743 and 8944 steps/day across study weeks. From this Australian study, it was observed that step counts were more successfully self-monitored than dietary choices. Similarly, in the present study, participants reported that the Fitbit was a favoured component of the SHIFT programme. Fitbits, along with similar commercially available wearable activity devices, and their associated apps facilitate several behaviour change techniques [45] and have been shown to have a favourable impact on activity levels from meta-analyses of controlled trials in adults (not specifically HGV drivers) over the short-term (3–6 months), leading to differences of + 951 steps/day between intervention and control groups [46].

No differences were observed between groups in anthropometric measures or markers of cardiometabolic health at 6 months. Interventions predominantly focusing on physical activity have been shown to have small to no effects on weight loss [47]. To have a bigger impact on weight, and measures of cardiometabolic risk, the SHIFT programme could be revised to include a greater emphasis on, and include ongoing support relating to, diet. Indeed, improved dietary quality was associated with weight loss in a lifestyle intervention with Finnish bus and truck drivers [48]. Furthermore, as highlighted by the Socioecological Model of Health [49], a wider intervention focus at multiple levels beyond the individual (for example, targeting dietary quality of foods available at rest stops) may improve the effectiveness of SHIFT.

A strength of this study was the implementation of a lifestyle health behaviour intervention within the workplace environment of an at-risk, underserved and hard-to-reach occupational group. The study involved 25 different transport sites operating within subcontracts across eight different industries. The range of industries represented by these sites, together with the demographic characteristics of our sample (mean age at baseline 48 years, 99% male, which matches exactly the characteristics of UK HGV drivers [14]), suggests that the included sample likely represents the 278,700 HGV drivers currently in employment [50]. Our intervention was evaluated through a fully powered, cluster RCT. The trial incorporated immediate (6 months) and longer-term (16–18 months) follow-up periods, and our primary outcome was a device-based measure, reducing the risk of bias associated with self-reported measures.

The COVID-19 pandemic had a large impact on the overall running of this trial. Whilst a strength of this study is the fact that we were able to follow-up participants at approximately 16–18 months following randomisation, once restrictions eased, the pandemic presents a major confounding factor which limits our ability to draw firm conclusions regarding the sustainability of the SHIFT programme. A further limitation was the high loss to follow-up experienced, which was beyond that initially predicted. We experienced a 31.4% loss-to-follow-up at the 6-month assessments, with the sample included in the primary outcome analysis reduced further (54.7% of the initial randomised sample) after taking into account activPAL compliance. Further losses to follow-up were experienced at the final follow-up, with 54% of the original sample attending this assessment. Multiple imputation to replace missing data suggested that the intervention effect in the complete case cohort may not be generalizable to the full cohort. We also lost 2 sites/clusters during the trial due to the collapse of their contracting companies. Transport managers highlighted high staff turnover rates within the industry during our process evaluation; indeed, the primary reason for non-completion of the trial was participants leaving their role. Future trials with this, or similar occupational groups, will need to account for potentially high loss-to-follow-up rates within sample size calculations, along with consideration of compliance rates to device-based measures, if appropriate. A final consideration within this trial is that although HGV drivers in the UK undergo a medical evaluation at 5-year intervals, the format of assessment and associated feedback may differ to that received in SHIFT. It is therefore possible that baseline assessments may have influenced the behaviour of drivers allocated to the control arm.

The high prevalence of drivers with obesity, along with the poor cardiometabolic health profile and sleep deprivation seen in our sample, highlight substantial health inequalities in this occupational group. Given the current, and increasing, shortfall of HGV drivers in the UK [17], the government and sector urgently need to address working conditions and the poor health profile of this ageing workforce. The already challenging working conditions are likely to be only exacerbated, as the low number of drivers have to compensate for driver shortages by expanding their own working hours, as relaxations in drivers’ hours rules have been re-introduced as a result of driver shortages, COVID-19 and Brexit [51]. Driver recruitment and a prioritisation on driver health is essential to combat the current challenges seen in maintaining critical supply chains and to support the UK’s economic recovery from the COVID-19 pandemic. Improving drivers’ health has significant implications, not only for the individual or their employer (through reductions in sickness absence and staff turnover), but also for the wider public through improving road safety for all users. Whilst all HGV drivers undertake compulsory Certificate of Professional Competence (CPC) training, this does not cover lifestyle health behaviours. The SHIFT programme, with ongoing development, has the potential to fill this void. Therefore, further work involving driver and stakeholder engagement is now needed to refine and translate SHIFT into a scalable CPC module, which should be evaluated over the longer-term to assess its impact in a real-world setting. Additionally, given the male dominated nature of HGV driving, the potential relevance of gender sensitivity within SHIFT should be explored further as part of this process. As obesity and sleep deprivation were highly prevalent in our sample, future research is also needed to better understand dietary eating practices and sleep management in this occupational group.

## Conclusions

The SHIFT programme led to a potentially clinically meaningful difference in daily steps, between trial arms, at 6 months. Whilst the longer-term impact is unclear, the programme offers potential to be incorporated into driver training courses to promote activity in this at-risk, underserved and hard-to-reach essential occupational group.

## Supplementary Information

**Additional file 1: Table S1.** Progression criteria results from the internal pilot. **Table S2.** Baseline Characteristics – completers vs. non-completers. Data are summarised as the median and inter-quartile range (IQR), unless otherwise stated. **Table S3.** Summary of key workday activPAL secondary outcome results from mixed effect linear regression models. **Table S4.** Summary of key non-workday activPAL secondary outcome results from mixed effect linear regression models. **Table S5.** Blood pressure measured at rest and during the mirror tracing task, at baseline and 6 months follow-up. Changes calculated from baseline are also presented. **Table S6.** Grip strength measured at baseline and 6 months follow-up, along with changes calculated from baseline. **Table S7.** Device-based measures of sleep outcomes from the GENEActiv at baseline and 6 months follow-up, along with changes calculated from baseline. **Table S8.** Reaction time from the Stroop Test measured at baseline and 6 months follow-up, along with changes calculated from baseline. **Table S9.** The prevalence of musculoskeletal discomfort reported in the past month for each body site, along with pain scores by body region, at baseline, 6 months and at the final follow-up. Changes calculated from baseline are also presented. **Table S10.** Anxiety, depression and social isolation scores measured at baseline, at 6 months and at the final follow-up, along with changes calculated from baseline. **Table S11.** Work-related psychosocial variables measured at baseline, at 6 months and at the final follow-up, along with changes calculated from baseline. **Table S12.** Markers of driving-related safety behaviour measured at baseline, at 6 months and at the final follow-up, along with changes calculated from baseline.

**Additional file 2.** CONSORT 2010 checklist of information to include when reporting a cluster randomised trial.

## Data Availability

The datasets used and/or analysed during the current study are available from the corresponding author on reasonable request.

## Abbreviations

BMI: Body mass index
BP: Blood pressure
CPC: Certificate of Professional Competence
DESMOND: Diabetes education and self-management for ongoing and newly diagnosed
HbA1c: Glycated haemoglobin
HDL cholesterol: High-density lipoprotein cholesterol
HGV: Heavy goods vehicle
ICC: Intraclass correlation coefficient
LDL cholesterol: Low-density lipoprotein cholesterol
IQR: Inter-quartile range
ITT: Intention to treat
LPA: Light intensity physical activity
MVPA: Moderate-vigorous physical activity
NHS: National Health Service
RCT: Randomised controlled trial
SD: Standard deviation
SHIFT: Structured Health Intervention For Truckers
UK: United Kingdom
USA: United States of America

## References

[1] Department for Transport (2020). Domestic Road Freight Statistics, United Kingdom 2019.

[2] Apostolopoulos Y, Sonmez S, Shattell MM, Belzer M (2010). Worksite-induced morbidities among truck drivers in the United States. AAOHN J.

[3] Sieber WK, Robinson CF, Birdsey J, Chen GX, Hitchcock EM, Lincoln JE, Nakata A, Sweeney MH (2014). Obesity and other risk factors: the national survey of U.S. long-haul truck driver health and injury. Am J Ind Med.

[4] Thiese MS, Hanowski RJ, Moffitt G, Kales SN, Porter RJ, Ronna B, Hartenbaum N, Hegmann KT (2018). A retrospective analysis of cardiometabolic health in a large cohort of truck drivers compared to the American working population. Am J Ind Med.

[5] Guest AJ, Chen YL, Pearson N, King JA, Paine NJ, Clemes SA (2020). Cardiometabolic risk factors and mental health status among truck drivers: a systematic review. BMJ Open.

[6] Hege A, Lemke MK, Apostolopoulos Y, Sonmez S (2018). Occupational health disparities among U.S. long-haul truck drivers: the influence of work organization and sleep on cardiovascular and metabolic disease risk. PLoS One.

[7] Abu Dabrh AM, Firwana B, Cowl CT, Steinkraus LW, Prokop LJ, Murad MH (2014). Health assessment of commercial drivers: a meta-narrative systematic review. BMJ Open.

[8] Mabry JE, Hosig K, Hanowski R, Zedalis D, Gregg J, Herbert WG (2016). Prevalence of metabolic syndrome in commercial truck drivers: a review. J Transp Health.

[9] Martin BC, Church TS, Bonnell R, Ben-Joseph R, Borgstadt T (2009). The impact of overweight and obesity on the direct medical costs of truck drivers. J Occup Environ Med.

[10] Thiese MS, Moffitt G, Hanowski RJ, Kales SN, Porter RJ, Hegmann KT (2015). Commercial driver medical examinations: prevalence of obesity, comorbidities, and certification outcomes. J Occup Environ Med.

[11] Thiese MS, Moffitt G, Hanowski RJ, Kales SN, Porter RJ, Hegmann KT (2015). Repeated cross-sectional assessment of commercial truck driver health. J Occup Environ Med.

[12] Crizzle A, Bigelow P, Adams D, Gooderham S, Myers A, Thiffault P (2017). Health and wellness of long-haul truck and bus drivers: a systematic literature review and directions for future research. J Transp Health.

[13] Office for National Statistics. Trend in life expectancy at birth and at age 65 by socio-economic position based on the National Statistics Socio-economic Classification, England and Wales: 1982—1986 to 2007—2011. Office for National Statistics; 2015.

[14] The Freight Transport Association. Logistics Report 2019, available at: https://www.santandercb.co.uk/sites/default/files/documents/fta_logistics_report_2019.pdf. accessed 6th Sept 2021.

[15] The All Party Parliamentary Group for Freight Transport. Barriers to youth employment in the freight transport sector, 2015, available at: http://www.fta.co.uk/export/sites/fta/_galleries/downloads/events/driver_crisis_delegate/mp_report_barriers_to_youth_employment.pdf, accessed 1st Aug, 2017.

[16] The Freight Transport Association. Logistics Report 2015. Freight Transport Association Limited; 2015, available at: http://www.fta.co.uk/export/sites/fta/_galleries/downloads/logistics_report/Web_files/LR15_WEB_270415.pdf, accessed 1st Aug 2017.

[17] RHA. A report on the driver shortage, 2021, available at: https://www.rha.uk.net/LinkClick.aspx?fileticket=ICI0CFWmVo%3d&portalid=0&timestamp=1627564639720, accessed 8th Sept 2021.

[18] Ng M, Yousuf B, Bigelow P, Van Eerd D (2015). Effectiveness of health promotion programmes for truck drivers: a systematic review. Health Educ J.

[19] Clemes SA, Varela Mato V, Munir F, Edwardson CL, Chen YL, Hamer M, Gray LJ, Bhupendra Jaicim N, Richardson G, Johnson V (2019). Cluster randomised controlled trial to investigate the effectiveness and cost-effectiveness of a Structured Health Intervention For Truckers (the SHIFT study): a study protocol. BMJ Open.

[20] Varela Mato V, Caddick N, King JA, Johnson V, Edwardson C, Yates T, Stensel DJ, Daly H, Nimmo MA, Clemes SA (2018). The impact of a novel Structured Health Intervention for Truckers (SHIFT) on physical activity and cardiometabolic risk factors. J Occup Environ Med.

[21] Bandura A (2004). Health promotion by social cognitive means. Health Educ Behav.

[22] Davies MJ, Heller S, Skinner TC, Campbell MJ, Carey ME, Cradock S, Dallosso HM, Daly H, Doherty Y, Eaton S (2008). Effectiveness of the diabetes education and self management for ongoing and newly diagnosed (DESMOND) programme for people with newly diagnosed type 2 diabetes: cluster randomised controlled trial. BMJ.

[23] Caddick N, Varela-Mato V, Nimmo MA, Clemes S, Yates T, King JA (2017). Understanding the health of lorry drivers in context: a critical discourse analysis. Health.

[24] Ryan CG, Grant PM, Tigbe WW, Granat MH (2006). The validity and reliability of a novel activity monitor as a measure of walking. Br J Sports Med.

[25] Winkler EA, Bodicoat DH, Healy GN, Bakrania K, Yates T, Owen N, Dunstan DW, Edwardson CL (2016). Identifying adults' valid waking wear time by automated estimation in activPAL data collected with a 24 h wear protocol. Physiol Meas.

[26] Edwardson C, Winkler E, Bodicoat DH, Yates T, Davies M, Dunstan D, Healy GN (2017). Considerations when using the activPAL monitor in field-based research with adult populations. J Sport Health Sci.

[27] Edwardson CL, Yates T, Biddle SJH, Davies MJ, Dunstan DW, Esliger DW, Gray LJ, Jackson B, O'Connell SE, Waheed G (2018). Effectiveness of the Stand More AT (SMArT) Work intervention: cluster randomised controlled trial. BMJ.

[28] Healy GN, Eakin EG, Owen N, Lamontagne AD, Moodie M, Winkler EA, Fjeldsoe BS, Wiesner G, Willenberg L, Dunstan DW (2016). A cluster randomized controlled trial to reduce office workers’ sitting time: effect on activity outcomes. Med Sci Sports Exerc.

[29] van Hees VT, Sabia S, Anderson KN, Denton SJ, Oliver J, Catt M, Abell JG, Kivimaki M, Trenell MI, Singh-Manoux A (2015). A novel, open access method to assess sleep duration using a wrist-worn accelerometer. PloS One.

[30] Sherry AP, Clemes SA, Chen YL, Edwardson C, Gray LJ, Guest A, King J, Rowlands AV, Ruettger K, Sayyah M (2022). Sleep duration and sleep efficiency in UK long-distance heavy goods vehicle drivers. Occup Environ Med..

[31] Friedewald WT, Levy RI, Fredrickson DS (1972). Estimation of the concentration of low-density lipoprotein cholesterol in plasma, without use of the preparative ultracentrifuge. Clin Chem.

[32] MacLeod CM (1991). Half a century of research on the Stroop effect: an integrative review. Psychol Bull.

[33] Feldman PJ, Cohen S, Lepore SJ, Matthews KA, Kamarck TW, Marsland AL (1999). Negative emotions and acute physiological responses to stress. Ann Behav Med.

[34] Guest AJ, Clemes SA, King JA, Chen YL, Ruettger K, Sayyah M, et al. Attenuated cardiovascular reactivity is related to higher anxiety and fatigue symptoms in truck drivers. Psychophysiology. 2021;58(9):e13872. 10.1111/psyp.13872. Epub 2021 Jun 4. 10.1111/psyp.13872 34086343

[35] Ewald B, Attia J, McElduff P (2014). How many steps are enough? Dose-response curves for pedometer steps and multiple health markers in a community-based sample of older Australians. J Phys Act Health.

[36] Thomson JL, Landry AS, Zoellner JM, Tudor-Locke C, Webster M, Connell C, Yadrick K (2012). Several steps/day indicators predict changes in anthropometric outcomes: HUB City Steps. BMC Public Health.

[37] Dwyer T, Ponsonby AL, Ukoumunne OC, Pezic A, Venn A, Dunstan D, Barr E, Blair S, Cochrane J, Zimmet P (2011). Association of change in daily step count over five years with insulin sensitivity and adiposity: population based cohort study. BMJ.

[38] Campbell MK, Fayers PM, Grimshaw JM (2005). Determinants of the intracluster correlation coefficient in cluster randomized trials: the case of implementation research. Clin Trials.

[39] Leyrat C, Morgan KE, Leurent B, Kahan BC (2018). Cluster randomized trials with a small number of clusters: which analyses should be used?. Int J Epidemiol..

[40] Rubin DB (1976). Inference and missing data. Biometrika.

[41] Rowlands A, Davies M, Dempsey P, Edwardson C, Razieh C, Yates T (2021). Wrist-worn accelerometers: recommending ~1.0 mg as the minimum clinically important difference (MCID) in daily average acceleration for inactive adults. Br J Sports Med.

[42] Lee IM, Shiroma EJ, Lobelo F, Puska P, Blair SN, Katzmarzyk PT (2012). Lancet Physical Activity Series Working Group. Effect of physical inactivity on major non-communicable diseases worldwide: an analysis of burden of disease and life expectancy. Lancet.

[43] Khunti K, Griffin S, Brennan A, Dallosso H, Davies MJ, Eborall HC, Edwardson CL, Gray LJ, Hardeman W, Heathcote L (2021). Promoting physical activity in a multi-ethnic population at high risk of diabetes: the 48-month PROPELS randomised controlled trial. BMC Med.

[44] Gilson ND, Pavey TG, Vandelanotte C, Duncan MJ, Gomersall SR, Trost SG, Brown WJ (2016). Chronic disease risks and use of a smartphone application during a physical activity and dietary intervention in Australian truck drivers. Aust N Z J Public Health.

[45] Duking P, Tafler M, Wallmann-Sperlich B, Sperlich B, Kleih S (2020). Behavior change techniques in wrist-worn wearables to promote physical activity: content analysis. JMIR mHealth uHealth.

[46] Ringeval M, Wagner G, Denford J, Pare G, Kitsiou S (2020). Fitbit-based interventions for healthy lifestyle outcomes: systematic review and meta-analysis. J Med Internet Res.

[47] Twells LK, Harris Walsh K, Blackmore A, Adey T, Donnan J, Peddle J, et al. Nonsurgical weight loss interventions: a systematic review of systematic reviews and meta-analyses. Obes Rev. 2021;22(11):e13320. 10.1111/obr.13320. Epub 2021 Aug 11. 10.1111/obr.13320 34378849

[48] Puhkala J, Kukkonen-Harjula K, Aittasalo M, Mansikkamaki K, Partinen M, Hublin C, Karmeniemi P, Sallinen M, Olkkonen S, Tokola K (2016). Lifestyle counseling in overweight truck and bus drivers - effects on dietary patterns and physical activity. Prev Med Reports.

[49] Raynor HA, Champagne CM (2016). Position of the academy of nutrition and dietetics: interventions for the treatment of overweight and obesity in adults. J Acad Nutr Diet..

[50] Department for Transport. Domestic Road Freight Statistics, United Kingdom 2020. National Statistics, 2021, available at: https://assets.publishing.service.gov.uk/government/uploads/system/uploads/attachment_data/file/1006792/domestic-road-freight-statistics-2020.pdf, accessed 8th Sept 2021

[51] Department for Transport and Driver & Vehicle Standards Agency. Coronavirus (COVID-19) and Brexit: guidance on drivers’ hours relaxations, available at: https://www.gov.uk/government/publications/covid-19-guidance-on-drivers-hours-relaxations/coronavirus-covid-19-guidance-on-drivers-hours-relaxations, accessed 6th Sept 2021.

