# Supplementary material for: The effectiveness of the Structured Health Intervention For Truckers (SHIFT): a cluster randomised controlled trial (RCT)
Source: BMC Med. 2022 May 24;20:195. doi: 10.1186/s12916-022-02372-7 (PMC9126630; doi:10.1186/s12916-022-02372-7)
Supplement: Supplementary file 1 — Additional file 1: Table S1. Progression criteria results from the internal pilot. Table S2. Baseline Characteristics – completers vs. non-completers. Data are summarised as the median and inter-quartile range (IQR), unless otherwise stated. Table S3. Summary of key workday activPAL secondary outcome results from mixed effect linear regression models. Table S4. Summary of key non-workday activPAL secondary outcome results from mixed effect linear regression models. Table S5. Blood pressure measured at rest and during the mirror tracing task, at baseline and 6 months follow-up. Changes calculated from baseline are also presented. Table S6. Grip strength measured at baseline and 6 months follow-up, along with changes calculated from baseline. Table S7. Device-based measures of sleep outcomes from the GENEActiv at baseline and 6 months follow-up, along with changes calculated from baseline. Table S8. Reaction time from the Stroop Test measured at baseline and 6 months follow-up, along with changes calculated from baseline. Table S9. The prevalence of musculoskeletal discomfort reported in the past month for each body site, along with pain scores by body region, at baseline, 6 months and at the final follow-up. Changes calculated from baseline are also presented. Table S10. Anxiety, depression and social isolation scores measured at baseline, at 6 months and at the final follow-up, along with changes calculated from baseline. Table S11. Work-related psychosocial variables measured at baseline, at 6 months and at the final follow-up, along with changes calculated from baseline. Table S12. Markers of driving-related safety behaviour measured at baseline, at 6 months and at the final follow-up, along with changes calculated from baseline. [file 12916_2022_2372_MOESM1_ESM.docx]

**Supplementary Tables**

**Table S1.** Progression criteria results from the internal pilot

| **Progression criteria** | **Observed outcome** |
| --- | --- |
| 1. All 24 sites required for the full sample size agree to take part in the study | 24 sites were identified and agreed to participate in the trial by November 2018. A 25^th^ site was recruited into the main trial phase, following agreement by the TSC, due to participants in one pilot site (a BP site) not being able to wear the activPAL during working hours for health and safety reasons. |
| 2. A minimum of 84 drivers agree to participate in the internal pilot | 98 drivers across the 6 internal pilot sites provided informed consent and participated in the baseline measures, of which 84% provided valid activPAL data at baseline. |
| 3. An average of 75% of drivers, randomised into the intervention arm, attended the education session across the 3 intervention depots | 74% of drivers in the intervention sites attended the education workshop. |
| 4. No more than 20% of participants fail to provide valid data for the primary outcome measure (activPAL-determined step counts) at baseline and at 6 months post randomisation, or withdraw/are lost to follow-up during the six-month intervention phase | Across the five sites completing the 6-month follow-up assessments by November 2018, 57% of participants provided valid activPAL data at baseline and follow-up.  *Strategies were discussed with, and approved by the TSC, for how activPAL compliance could be improved for the main trial phase.* |

**Table S2**. Baseline Characteristics – completers vs. non-completers. Data are summarised as the median and inter-quartile range (IQR), unless otherwise stated.

| Characteristics | Control | | SHIFT | | Total | |
| --- | --- | --- | --- | --- | --- | --- |
|  | Non-Completers  (n=69) | Completers^a^  (n=130) | Non-Completers  (n=83) | Completers  (n=100) | Non-Completers (n=152) | Completers  (n=230) |
| Cluster size, n (%) |  |  |  |  |  |  |
| Small | 31 (44.9%) | 50 (38.5%) | 52 (62.7%) | 41 (41.0%) | 83 (54.6%) | 91 (39.6%) |
| Large | 38 (55.1%) | 80 (61.5%) | 31 (37.4%) | 59 (59.0%) | 69 (45.4%) | 139 (60.4%) |
|  |  |  |  |  |  |  |
| Demographics |  |  |  |  |  |  |
| Age (years) | 49.95  (42.50, 56.41) | 49.25  (40.64, 55.16) | 49.82  (43.77, 54.96) | 50.04  (41.66, 55.40) | 49.82  (43.07, 55.47) | 49.45  (41.12, 55.24) |
| Number of years as a HGV driver | 19.50  (11.33, 28.25) | 12.34  (5.17, 24.02) | 17.50  (9.00, 25.83) | 17.00  (10.50, 25.00) | 17.88  (10.00, 27.00) | 14.50  (7.38, 25.00) |
|  |  |  |  |  |  |  |
| Biometric Measures |  |  |  |  |  |  |
| Body mass Index (kg/m^2^) | 30.57  (27.86, 32.84) | 28.89  (26.64, 32.90) | 29.58  (26.56, 33.54) | 30.03  (26.93, 34.30) | 30.06  (27.08, 32.96) | 29.71  (26.87, 33.47) |
| activPAL variables |  |  |  |  |  |  |
| Number of steps at baseline (steps/day) | 7969.4  (6718.7, 9894.9) | 8579.5  (6920.0, 10327.0) | 8813.6  (7208.6, 11973.3) | 8605.5  (6978.6, 11067.7) | 8531.7  (6879.8, 10678.6) | 8597.5  (6964.9, 10695.7) |

^a^ Defined as providing valid activPAL data at 6 months

**Table S3.** Summary of key workday activPAL secondary outcome results from mixed effect linear regression models

| Daily Variables (min/day) | Number of clusters | | Number of participants | | Baseline  Mean (SD) | | Follow-up  Mean (SD) | | Mean change from baseline to Follow-up (SD)^a^ | | SHIFT vs. Control | |
| --- | --- | --- | --- | --- | --- | --- | --- | --- | --- | --- | --- | --- |
|  | **Control** | **SHIFT** | **Control** | **SHIFT** | **Control** | **SHIFT** | **Control** | **SHIFT** | **Control** | **SHIFT** | **Adjusted mean difference**  **(95% CI) ^a^** | **P-value** |
| Steps per day ^b^ |  |  |  |  |  |  |  |  |  |  |  |  |
| 6 months | 13 | 12 | 111 | 84 | 9308 (3154) | 9547 (3458) | 8890 (3041) | 9357 (3241) | -418 (2046) | -190 (2649) | 541 (-269, 1351) | 0.190 |
| 16-18 months | 12 | 10 | 88 | 66 | 9394 (3177) | 9881 (3472) | 9491 (3388) | 9456 (3390) | 97 (2817) | -425 (3067) | -325 (-1578, 928) | 0.611 |
| Time sitting ^b^ |  |  |  |  |  |  |  |  |  |  |  |  |
| 6 months | 13 | 12 | 111 | 84 | 720 (95) | 713 (118) | 740 (94) | 700 (96) | 20 (83.41) | -13 (101) | -14 (-36, 8) | 0.215 |
| 16-18 months | 12 | 10 | 88 | 66 | 718 (98) | 701 (122) | 726 (117) | 707 (91) | 8 (97) | 6 (115) | 0.1 (-22, 22) | 0.995 |
| Time standing ^b^ |  |  |  |  |  |  |  |  |  |  |  |  |
| 6 months | 13 | 12 | 111 | 84 | 191 (58) | 195 (61) | 186 (52) | 194 (59) | -5 (52) | -2 (60) | 10 (-3, 23) | 0.129 |
| 16-18 months | 12 | 10 | 88 | 66 | 191 (57) | 201 (68) | 190 (60) | 195 (48) | -2 (55) | -6 (64) | 3 (-12, 18) | 0.708 |
| Time stepping ^b^ |  |  |  |  |  |  |  |  |  |  |  |  |
| 6 months | 13 | 12 | 111 | 84 | 120 (37) | 124 (43) | 115 (37) | 123 (42) | -5 (24) | -2 (30) | 7 (-3, 16) | 0.162 |
| 16-18 months | 12 | 10 | 88 | 66 | 122 (37) | 128 (43) | 122 (40) | 122 (42) | 0.05 (32) | -5 (31) | -3 (-17, 10) | 0.621 |
| Time LPA ^b^ |  |  |  |  |  |  |  |  |  |  |  |  |
| 6 months | 13 | 12 | 111 | 84 | 105 (35) | 110 (38) | 102 (34) | 109 (40) | -3 (22) | -1 (26) | 4 (-5, 13) | 0.343 |
| 16-18 months | 12 | 10 | 88 | 66 | 109 (33) | 112 (39) | 108 (36) | 108 (39) | -1 (27) | -4 (24) | -2 (-13, 8) | 0.692 |
| Time MVPA ^b^ |  |  |  |  |  |  |  |  |  |  |  |  |
| 6 months | 13 | 12 | 111 | 84 | 14 (13) | 14 (13) | 13 (10) | 14 (11) | -2 (10) | -0.1 (14) | 2 (-2, 6) | 0.357 |
| 16-18 months | 12 | 10 | 88 | 66 | 13 (11) | 16 (13) | 14 (11) | 15 (16) | 1 (10) | -1 (19) | -0.4 (-5, 5) | 0.875 |

^a^ Adjusted for variable at baseline, average waking wear time across baseline and 6 (or 12) months, and cluster size category (Small <40; Large ≥40) with a random effect for cluster (depot).

^b^ ≥1 valid day at baseline and 6 (or 16-18) months

**Table S4.** Summary of key non-workday activPAL secondary outcome results from mixed effect linear regression models

| Daily Variables (min/day) | Number of clusters | | Number of participants | | Baseline  Mean (SD) | | Follow-up  Mean (SD) | | Mean change from baseline to Follow-up (SD) | | SHIFT vs. Control | |
| --- | --- | --- | --- | --- | --- | --- | --- | --- | --- | --- | --- | --- |
|  | **Control** | **SHIFT** | **Control** | **SHIFT** | **Control** | **SHIFT** | **Control** | **SHIFT** | **Control** | **SHIFT** | **Adjusted mean difference**  **(95% CI) ^a^** | **P-value** |
| Steps per day ^b^ |  |  |  |  |  |  |  |  |  |  |  |  |
| 6 months | 13 | 12 | 102 | 77 | 8467 (5248) | 8733 (3894) | 6897 (3331) | 9077 (4895) | -1570 (4754) | 344 (4150) | 2012 (480, 3545) | 0.010 |
| 16-18 months | 12 | 9 | 81 | 65 | 8348 (5935) | 9252 (3994) | 7397 (4116) | 9096 (4167) | -951 (5561) | -156 (4031) | 1392 (-277, 3060) | 0.102 |
| Time sitting ^b^ |  |  |  |  |  |  |  |  |  |  |  |  |
| 6 months | 13 | 12 | 102 | 77 | 577 (118) | 587 (121) | 610 (131) | 584 (132) | 33 (110) | -4 (123) | -40 (-65, -14) | 0.003 |
| 16-18 months | 12 | 9 | 81 | 65 | 585 (122) | 568 (105) | 595 (140) | 563 (114) | 11 (116) | -5 (112) | -20 (-62, 23) | 0.360 |
| Time standing ^b^ |  |  |  |  |  |  |  |  |  |  |  |  |
| 6 months | 13 | 12 | 102 | 77 | 233 (75) | 234 (73) | 214 (88) | 240 (88) | -18 (67) | 6 (82) | 20 (-1, 41) | 0.059 |
| 16-18 months | 12 | 9 | 81 | 65 | 222 (75) | 243 (69) | 213 (84) | 230 (74) | -9 (68) | -12 (81) | 7 (-22, 36) | 0.630 |
| Time stepping ^cb^ |  |  |  |  |  |  |  |  |  |  |  |  |
| 6 months | 13 | 12 | 102 | 77 | 110 (54) | 114 (42) | 93 (40) | 117 (51) | -17 (48) | 3 (44) | 21 (6, 37) | 0.008 |
| 16-18 months | 12 | 9 | 81 | 65 | 109 (62) | 120 (42) | 100 (47) | 117 (47) | -9 (54) | -3 (47) | 14 (-5, 32) | 0.155 |
| Time LPA ^b^ |  |  |  |  |  |  |  |  |  |  |  |  |
| 6 months | 13 | 12 | 102 | 77 | 93 (36) | 96 (31) | 81 (33) | 94 (35) | -12 (31) | -2 (31) | 9 (2, 17) | 0.017 |
| 16-18 months | 12 | 9 | 81 | 65 | 92 (42) | 101 (32) | 87 (39) | 97 (40) | -5 (27) | -3 (40) | 5.28 (-7, 17) | 0.381 |
| Time MVPA ^b^ |  |  |  |  |  |  |  |  |  |  |  |  |
| 6 months | 13 | 12 | 102 | 77 | 17 (37) | 18 (21) | 12 (14) | 23 (29) | -6 (37) | 4 (29) | 11 (1, 20) | 0.027 |
| 16-18 months | 12 | 9 | 81 | 65 | 17 (42) | 19 (22) | 13 (21) | 20 (21) | -4 (45) | 0.5 (18) | 6 (-2, 14) | 0.123 |

^a^ Adjusted for variable at baseline, average waking wear time across baseline and 6 (or 12) months, and cluster size category (Small <40; Large ≥40) with a random effect for cluster (depot).

^b^ ≥1 valid day at baseline and 6 (or 12) months.

**Table S5.** Blood pressure measured at rest and during the mirror tracing task, at baseline and 6 months follow-up. Changes calculated from baseline are also presented

| Variable | Number of participants | | Baseline  Median (IQR) | | Follow-up  Median (IQR) | | Median change from baseline to 6 months follow-up (IQR) | |
| --- | --- | --- | --- | --- | --- | --- | --- | --- |
|  | **Control** | **SHIFT** | **Control** | **SHIFT** | **Control** | **SHIFT** | **Control** | **SHIFT** |
| Resting blood pressure (BP) and heart rate^a^ | | | | | | |  |  |
| Systolic BP (mmHg) | 145 | 104 | 130  (120, 139) | 130  (122, 138) | 127  (118, 135) | 127  (118, 136) | -2  (-9, 4) | -3  (-10, 5) |
| Diastolic BP (mmHg) | 145 | 104 | 82  (76, 88) | 82  (78, 88) | 80  (74, 86) | 80  (75, 87) | -2  (-6, 4) | -1  (-6, 4) |
| Heart rate (bpm | 143 | 104 | 67  (60, 74) | 68  (60, 74) | 66  (60, 73) | 66  (58, 72) | 0  (-5, 5) | -1  (-6, 3) |
| Blood pressure and heart rate during the mirror tracing task^b^ | | | | | | |  |  |
| Systolic BP (mmHg) | 138 | 100 | 146  (134, 159) | 145  (137, 158) | 142  (132, 156) | 143  (131, 153) | -3  (-12, 5) | -4  (-10, 4) |
| Diastolic BP (mmHg) | 138 | 100 | 91  (85, 98) | 92  (85, 103) | 89  (81, 95) | 89  (84, 96) | -2  (-9, 3) | -3  (-9, 1) |
| Heart rate (bpm | 136 | 100 | 74  (67, 80) | 72  (66, 82) | 72  (66, 78) | 72  (65, 78) | -1  (-7, 4) | -2  (-7, 3) |
| Psychophysiological reactivity^c^ | | | | | | |  |  |
| Δ Systolic BP (mmHg) | 138 | 100 | 17  (10, 22) | 16  (9, 26) | 16  (9, 24) | 15  (8, 22) | -1  (-8, 8) | -3  (-9, 6) |
| Δ Diastolic BP (mmHg) | 138 | 100 | 10  (6, 14) | 10  (7, 14) | 8  (4, 12) | 8  (4, 14) | -2  (-6, 4) | -1  (-6, 3) |
| Δ Heart rate (bpm) | 136 | 100 | 7  (2, 10) | 6  (3, 10) | 5  (2, 8) | 6  (4, 10) | -1  (-6, 2) | 0  (-4, 5) |

^a^ Three measures of resting blood pressure and heart rate were taken after a 20-minute rest period, the average of the second and third measures were calculated for each participant.

^b^ Two measures of blood pressure and heart rate were taken during the mirror tracing task, the average of these measures was calculated for each participant.

^c^ To calculate reactivity to stress, the mean systolic and diastolic blood pressure and heart rate values recorded at rest were subtracted from the corresponding mean values recorded during the mirror tracing task for each participant.

**Table S6.** Grip strength measured at baseline and 6 months follow-up, along with changes calculated from baseline

| Variable | Number of participants | | Baseline  Mean (SD) | | Follow-up  Mean (SD) | | Mean change from baseline to 6 months follow-up (SD) | |
| --- | --- | --- | --- | --- | --- | --- | --- | --- |
|  | **Control** | **SHIFT** | **Control** | **SHIFT** | **Control** | **SHIFT** | **Control** | **SHIFT** |
| Grip strength | | | | | | |  |  |
| Right hand (kg) | 144 | 104 | 52.0  (9.6) | 51.9  (8.5) | 52.2  (10.2) | 52.7  (8.9) | 0.2  (6.9) | 0.8  (6.2) |
| Left hand (kg) | 144 | 104 | 50.0  (9.2) | 49.1  (7.5) | 50.0  (9.8) | 50.6  (8.0) | 0.02  (6.6) | 1.5  (5.1) |
| Average (kg) | 143 | 104 | 51.0  (9.0) | 50.5  (7.6) | 51.0  (9.5) | 51.6  (7.9) | 0.09  (5.7) | 1.1  (4.9) |

**Table S7.** Device-based measures of sleep outcomes from the GENEActiv at baseline and 6 months follow-up, along with changes calculated from baseline

| Variable | Number of participants | | Baseline  Mean (SD) | | Follow-up  Mean (SD) | | Mean change from baseline to 6 months follow-up (SD) | |
| --- | --- | --- | --- | --- | --- | --- | --- | --- |
|  | **Control** | **SHIFT** | **Control** | **SHIFT** | **Control** | **SHIFT** | **Control** | **SHIFT** |
| All days | | | | | | |  |  |
| Number of valid nights | 118 | 89 | 6 (1) | 5 (2) | 5 (1) | 5 (1) | -1 (1) | 0 (2) |
| Sleep window^a^ duration (mins) | 118 | 89 | 425 (54) | 419 (54) | 410 (62) | 405 (65) | -15 (70) | -14 (58) |
| Sleep duration (mins) | 118 | 89 | 370 (54) | 368 (55) | 355 (57) | 357 (59) | -15 (57) | -11 (51) |
| Sleep efficiency^b^ (%) | 118 | 89 | 87 (7) | 88 (6) | 87 (6) | 89 (6) | 0 (4) | 1 (5) |
| Workdays | | | | | | |  |  |
| Number of valid nights | 100 | 67 | 3 (1) | 3 (1) | 3 (1) | 3 (1) | 0 (1) | 0 (2) |
| Sleep window^a^ duration (mins) | 100 | 67 | 420 (61) | 402 (67) | 363 (74) | 369 (79) | -57 (80) | -33 (70) |
| Sleep duration (mins) | 100 | 67 | 366 (56) | 354 (65) | 317 (68) | 329 (72) | -49 (69) | -25 (64) |
| Sleep efficiency^b^ (%) | 100 | 67 | 87 (8) | 88 (7) | 88 (7) | 89 (6) | 1 (6) | 1 (5) |
| Non-workdays | | | | | | |  |  |
| Number of valid nights | 96 | 60 | 2 (1) | 2 (1) | 2 (1) | 2 (1) | 0 (1) | 0 (1) |
| Sleep window^a^ duration (mins) | 96 | 60 | 422 (79) | 429 (78) | 482 (92) | 462 (103) | 60 (107) | 33 (123) |
| Sleep duration (mins) | 96 | 60 | 367 (74) | 377 (78) | 416 (78) | 406 (95) | 49 (90) | 29 (111) |
| Sleep efficiency^b^ (%) | 96 | 60 | 87 (7) | 88 (7) | 87 (7) | 88 (8) | 0 (6) | 0 (5) |

**^a^** Sleep window was defined as the time between ‘lights out’ and out of bed time.

^b^ Sleep efficiency range: 0 – 100%, with higher value indicating better sleep efficiency

**Table S8.** Reaction time from the Stroop Test measured at baseline and 6 months follow-up, along with changes calculated from baseline

| Variable | Number of participants | | Baseline  Median (IQR) | | Follow-up  Median (IQR) | | Median change from baseline to 6 months follow-up (IQR) | |
| --- | --- | --- | --- | --- | --- | --- | --- | --- |
|  | **Control** | **SHIFT** | **Control** | **SHIFT** | **Control** | **SHIFT** | **Control** | **SHIFT** |
| Reaction time (ms) | | | | | | |  |  |
| Congruent condition^a^ | 111 | 68 | 988  (880, 1112) | 998  (888, 1110) | 959  (895, 1058) | 976  (878, 1058) | -17  (-104, 68) | 4  (-102, 59) |
| Incongruent condition^b^ | 111 | 68 | 1121  (992, 1325) | 1125  (994, 1420) | 1078  (977, 1247) | 1095  (968, 1268) | -41  (-145, 36) | -44  (-135, 43) |

^a^ naming colour of font of random words

^b^ naming colour of font of colour names, written in a different font colour

**Table S9.** The prevalence of musculoskeletal discomfort reported in the past month for each body site, along with pain scores by body region, at baseline, 6 months and at the final follow-up. Changes calculated from baseline are also presented.

| Prevalence of musculoskeletal discomfort in the past month per body area^a^ | Number of participants | | Baseline  Proportion (%) | | Follow-up  Proportion (%) | | Change in proportion (%) | |
| --- | --- | --- | --- | --- | --- | --- | --- | --- |
|  | **Control** | **SHIFT** | **Control** | **SHIFT** | **Control** | **SHIFT** | **Control** | **SHIFT** |
| Neck |  |  |  |  |  |  |  |  |
| 6 months | 145 | 112 | 39% | 34% | 36% | 34% | -3% | 0% |
| 16-18 months | 101 | 91 | 42% | 31% | 38% | 37% | -4% | 7% |
| Shoulder |  |  |  |  |  |  |  |  |
| 6 months | 145 | 112 | 40% | 44% | 41% | 43% | 1% | -1% |
| 16-18 months | 101 | 91 | 44% | 42% | 49% | 43% | 5% | 1% |
| Upper back |  |  |  |  |  |  |  |  |
| 6 months | 145 | 112 | 21% | 27% | 17% | 20% | -4% | -7% |
| 16-18 months | 101 | 91 | 22% | 29% | 25% | 26% | 3% | -2% |
| Elbow |  |  |  |  |  |  |  |  |
| 6 months | 145 | 112 | 21% | 19% | 19% | 24% | -2% | 5% |
| 16-18 months | 101 | 91 | 23% | 15% | 29% | 18% | 6% | 2% |
| Wrist/hand |  |  |  |  |  |  |  |  |
| 6 months | 145 | 112 | 26% | 33% | 29% | 31% | 3% | -2% |
| 16-18 months | 101 | 91 | 30% | 34% | 38% | 33% | 8% | -1% |
| Lower back |  |  |  |  |  |  |  |  |
| 6 months | 145 | 112 | 57% | 56% | 50% | 49% | -7% | -7% |
| 16-18 months | 101 | 91 | 59% | 57% | 53% | 47% | -6% | -10% |
| Hip/thigh |  |  |  |  |  |  |  |  |
| 6 months | 145 | 112 | 26% | 24% | 14% | 13% | -12% | -11% |
| 16-18 months | 101 | 91 | 24% | 25% | 22% | 22% | -2% | -3% |
| Knee |  |  |  |  |  |  |  |  |
| 6 months | 145 | 112 | 45% | 44% | 41% | 40% | -3% | -4% |
| 16-18 months | 101 | 91 | 47% | 48% | 42% | 42% | -5% | -7% |
| Ankle/feet |  |  |  |  |  |  |  |  |
| 6 months | 145 | 112 | 28% | 27% | 21% | 21% | -8% | -5% |
| 16-18 months | 101 | 91 | 32% | 26% | 29% | 29% | -3% | 2% |
| Discomfort scores | **Number of participants** | | **Baseline**  **Median (IQR)** | | **Follow-up**  **Median (IQR)** | | **Median change from baseline to follow-up (IQR)** | |
|  | **Control** | **SHIFT** | **Control** | **SHIFT** | **Control** | **SHIFT** | **Control** | **SHIFT** |
| Upper extremity discomfort^b^ | | | | | | | | |
| 6 months | 145 | 112 | 0.5  (0.0, 1.5) | 1.0  (0.0, 2.0) | 0.5  (0.0, 1.8) | 0.8  (0.0, 2.0) | 0.0  (-0.5, 0.5) | 0.0  (-0.5, 0.5) |
| 16-18 months | 101 | 91 | 0.5  (0.0, 1.5) | 0.8  (0.0, 1.9) | 1.0  (0.0, 2.5) | 0.8  (0.0, 2.0) | 0.0  (-0.3, 1.0) | 0.0  (-0.5, 0.6) |
| Lower extremity discomfort^c^ | | | | | | | | |
| 6 months | 145 | 112 | 0.7  (0.0, 2.0) | 0.3  (0.0, 1.7) | 0.3  (0.0, 1.7) | 0.3  (0.0, 1.7) | 0.0  (-1.0, 0.3) | 0.0  (-0.3, 0.3) |
| 16-18 months | 101 | 91 | 1.0  (0.0, 2.0) | 0.7  (0.0, 2.0) | 0.3  (0.0, 1.7) | 0.3  (0.0, 2.0) | 0.0  (-0.7, 0.3) | 0.0  (-0.3, 0.7) |
| Overall discomfort^d^ | | | | | | | | |
| 6 months | 145 | 112 | 1.0  (0.3, 1.8) | 1.0  (0.3, 1.9) | 0.9  (0.2, 1.8) | 0.9  (0.3, 1.7) | 0.0  (-0.7, 0.4) | 0.0  (-0.6, 0.3) |
| 16-18 months | 101 | 91 | 1.1  (0.4, 1.7) | 1.0  (0.4, 1.9) | 1.3  (0.3, 2.2) | 1.0  (0.2, 2.1) | 0.0  (-0.3, 0.8) | 0.0  (-0.4, 0.6) |

^a^ Standardised Nordic Questionnaire, participants reported trouble (such as aches, pain, discomfort, numbness) in 9 body areas occurring in the past month on a 11-point Likert scale ranging from 0 ‘no trouble’ to 10 ‘severe trouble’.

^b^ Upper extremity discomfort was calculated by averaging discomfort ratings from the shoulder, upper back, elbow and wrist/hand.

^c^ Lower extremity discomfort was calculated by averaging discomfort ratings from the hip, knee and ankle/feet.

d Overall discomfort was calculated by averaging discomfort ratings from all nine areas.

**Table S10.** Anxiety, depression and social isolation scores measured at baseline, at 6 months and at the final follow-up, along with changes calculated from baseline

| Variable | Number of participants | | Baseline  Median (IQR) | | Follow-up  Median (IQR) | | Median change from baseline to follow-up (IQR) | |
| --- | --- | --- | --- | --- | --- | --- | --- | --- |
|  | **Control** | **SHIFT** | **Control** | **SHIFT** | **Control** | **SHIFT** | **Control** | **SHIFT** |
| HADS Anxiety^a^ | | | | | | |  |  |
| 6 months | 145 | 113 | 5  (3, 7) | 4  (2, 7) | 4  (2, 6) | 4  (2, 7) | -1  (-3, 1) | 0  (-2, 1) |
| 16-18 months | 100 | 88 | 5  (3, 7) | 4  (2, 7) | 4  (2, 7) | 4  (2, 7) | -1  (-2, 1) | 0  (-1, 1) |
| HADS Depression^a^ | | | | | | | | |
| 6 months | 145 | 113 | 3  (2, 7) | 3  (1, 5) | 3  (1, 6) | 3  (1, 5) | -1  (-2, 1) | 0  (-1, 1) |
| 16-18 months | 100 | 88 | 3  (2, 6) | 3  (1, 4) | 3  (1, 6) | 3  (1, 5) | 0  (-2, 1) | 0  (-1, 1) |
| Social isolation^b^ | | | | | | | | |
| 6 months | 145 | 113 | 44  (39, 51) | 41  (34, 49) | 44  (34, 49) | 43  (34, 50) | 0  (-5, 2) | 0  (0, 5) |
| 16-18 months | 101 | 88 | 44  (39, 51) | 41  (34, 49) | 47  (39, 52) | 44  (34, 51) | 0  (-2, 4) | 0  (0, 5) |

^a^ HADS Anxiety and Depression scores range from 0 to 21, with higher scores indicating a greater degree of anxiety/depression, a score of 7 or less is classified as ‘no symptoms’.

^b^ Social isolation scale score range from 33.9 to 76.9, with higher scores indicating a greater perception of social isolation.

**Table S11.** Work-related psychosocial variables measured at baseline, at 6 months and at the final follow-up, along with changes calculated from baseline

| Variable | Number of participants | | Baseline  Median (IQR) | | Follow-up  Median (IQR) | | Median change from baseline to follow-up (IQR) | |
| --- | --- | --- | --- | --- | --- | --- | --- | --- |
|  | **Control** | **SHIFT** | **Control** | **SHIFT** | **Control** | **SHIFT** | **Control** | **SHIFT** |
| Utrecht Work Engagement Scale (UWES)^a^ | | | | | | |  |  |
| Vigour | | | | | | |  |  |
| 6 months | 144 | 113 | 4.0  (3.3, 5.0) | 4.0  (3.0, 4.7) | 4.0  (3.0, 5.0) | 4.0  (2.7, 5.0) | 0.0  (-0.7, 0.3) | 0.0  (-0.7, 0.3) |
| 16-18 months | 100 | 89 | 4.0  (3.3, 5.0) | 3.7  (3.0, 4.7) | 4.3  (3.3, 4.7) | 3.7  (2.3, 4.7) | 0.0  (-0.7, 0.7) | -0.3  (-0.7, 0.0) |
| Dedication | | | | | | | | |
| 6 months | 144 | 113 | 4.3  (3.3, 5.3) | 4.0  (3.0, 5.0) | 4.3  (3.3, 5.0) | 4.3  (3.0, 5.0) | 0.0  (-0.7, 0.3) | 0.0  (-0.3, 0.7) |
| 16-18 months | 100 | 89 | 3.7  (4.7, 6.0) | 4.0  (3.0, 5.0) | 4.3  (3.6, 5.0) | 3.7  (2.7, 5.0) | -0.2  (-0.7, 0.3) | 0.0  (-0.7, 0.3) |
| Absorption | | | | | | | | |
| 6 months | 144 | 113 | 4.0  (3.0, 5.0) | 3.7  (2.7, 4.7) | 3.7  (2.7, 4.7) | 4.0  (2.7, 4.7) | 0.0  (-0.7, 0.3) | 0.0  (-0.7, 0.7) |
| 16-18 months | 100 | 89 | 3.3  (3.7, 5.0) | 3.7  (2.3, 4.7) | 4.0  (3.3, 5.0) | 3.7  (2.3, 4.7) | 0.0  (-1.0, 0.7) | 0.0  (-0.7, 0.3) |
| Overall Summary score | | | | | | | | |
| 6 months | 144 | 113 | 4.3  (3.2, 5.0) | 3.9  (3.0, 4.9) | 4.1  (3.0, 4.8) | 3.9  (2.8, 4.8) | -0.1  (-0.7, 0.3) | 0.0  (-0.4, 0.4) |
| 16-18 months | 100 | 89 | 3.7  (4.4, 5.2) | 3.8  (2.9, 4.9) | 4.2  (3.4, 4.8) | 3.7  (2.7, 4.6) | -0.1  (-0.7, 0.6) | -0.1  (-0.7, 0.3) |
| Occupational Fatigue Exhaustion Recovery (OFER 15) Scale^b^ | | | | | | | | |
| Chronic Fatigue | | | | | | | | |
| 6 months | 144 | 113 | 33.3  (16.7, 60.8) | 33.3  (16.7, 53.3) | 36.7  (16.7, 56.7) | 36.7  (16.7, 56.7) | 0.0  (-6.7, 10.8) | 0.0  (-10.0, 13.3) |
| 16-18 months | 100 | 90 | 31.7  (16.7, 53.3) | 31.7  (16.7, 53.3) | 36.7  (20.0, 57.5) | 33.3  (20.0, 53.3) | 3.3  (-3.3, 13.3) | 1.7  (-13.3, 16.7) |
| Acute Fatigue | | | | | | | | |
| 6 months | 144 | 113 | 46.7  (32.5, 60.0) | 50.0  (30.0, 63.3) | 43.3  (26.7, 63.3) | 46.7  (30.0, 66.7) | 0.0  (-10.8, 10.0) | 0.0  (-13.3, 6.7) |
| 16-18 months | 100 | 90 | 50.0  (33.3, 60.8) | 48.3  (26.7, 63.3) | 50.0  (30.0, 63.3) | 50.0  (30.8, 66.7) | -3.3  (-14.2, 13.3) | 3.3  (-6.7, 13.3) |
| Inter-shift Recovery | | | | | | | | |
| 6 months | 144 | 113 | 55.0  (40.0, 80.0) | 60.0  (43.3, 76.7) | 60.0  (43.3, 76.7) | 56.7  (43.3, 76.7) | 0.0  (-13.3, 10.0) | 0.0  (-10.0, 6.7) |
| 16-18 months | 100 | 90 | 53.3  (42.5, 80.0) | 60.0  (43.3, 80.0) | 56.7  (40.0, 77.5) | 53.3  (40.8, 80.0) | 0.0  (-10.0, 10.0) | 0.0  (-10.0, 12.5) |
| Job Satisfaction rating^c^ | | | | | | | | |
| 6 months | 144 | 113 | 5.0  (4.0, 6.0) | 5.0  (4.0, 6.0) | 5.0  (4.0, 6.0) | 5.0  (4.0, 6.0) | 0.0  (-1.0, 0.0) | 0.0  (-1.0, 0.0) |
| 16-18 months | 100 | 90 | 6.0  (4.0, 6.0) | 5.0  (4.0, 6.0) | 5.0  (4.0, 6.0) | 5.0  (4.0, 6.0) | 0.0  (-1.0, 0.0) | 0.0  (-1.0, 1.0) |
| Job Performance rating^c^ | | | | | | | | |
| 6 months | 144 | 113 | 6.0  (5.0, 7.0) | 6.0  (6.0, 7.0) | 6.0  (6.0, 7.0) | 6.0  (6.0, 7.0) | 0.0  (0.0, 0.0) | 0.0  (0.0, 0.0) |
| 16-18 months | 100 | 90 | 6.0  (5.0, 6.3) | 6.0  (6.0, 7.0) | 6.0  (5.0, 7.0) | 6.0  (6.0, 7.0) | 0.0  (-1.0, 1.0) | 0.0  (0.0, 0.0) |
| Sickness absence (days)^d^ | | | | | | | | |
| 6 months | 142 | 113 | 0.0  (0.0, 2.0) | 0.0  (0.0, 1.0) | 0.0  (0.0, 3.0) | 0.0  (0.0, 0.0) | 0.0  (0.0, 0.0) | 0.0  (0.0, 0.0) |
| 16-18 months | 100 | 90 | 0.0  (0.0, 2.0) | 0.0  (0.0, 0.0) | 0.0  (0.0, 0.0) | 0.0  (0.0, 0.0) | 0.0  (-1.3, 0.0) | 0.0  (0.0, 0.0) |
| Presenteeism (days)^d^ | | | | | | | | |
| 6 months | 141 | 113 | 2.0  (0.0, 5.0) | 2.0  (0.0, 5.0) | 1.0  (0.0, 5.0) | 1.0  (0.0, 5.0) | 0.0  (-2.0, 1.0) | 0.0  (-1.3, 3.0) |
| 16-18 months | 98 | 89 | 2.0  (0.0, 5.0) | 2.0  (0.0, 5.0) | 0.0  (0.0, 2.0) | 0.0  (0.0, 5.0) | 0.0  (-4.0, 0.0) | 0.0  (-2.0, 2.0) |
| Workability rating^e^ | | | | | | | | |
| 6 months | 144 | 113 | 9.0  (8.0, 10.0) | 8.5  (8.0, 10.0) | 8.0  (7.0, 9.0) | 9.0  (8.0, 9.0) | 0.0  (-1.0, 1.0) | 0.0  (-1.0, 1.0) |
| 16-18 months | 96 | 87 | 9.0  (8.0, 9.0) | 9.0  (8.0, 10.0) | 8.0  (8.0, 9.0) | 9.0  (8.0, 10.0) | 0.0  (-1.0, 0.0) | 0.0  (-1.0, 1.0) |
| Work Demands (Health and Safety Executive Management Standards Indicator Tool)^f^ | | | | | | | | |
| Demand summary score | | | | | | | | |
| 6 months | 144 | 113 | 2.1  (1.8, 2.5) | 2.1  (1.6, 2.9) | 2.1  (1.8, 2.6) | 2.1  (1.6, 2.6) | 0.1  (-0.3, 0.4) | 0.0  (-0.4, 0.3) |
| 16-18 months | 100 | 90 | 2.1  (1.8, 2.7) | 2.2  (1.6, 2.9) | 2.2  (1.6, 2.8) | 2.3  (1.6, 2.8) | 0.1  (-0.4, 0.5) | 0.0  (-0.5, 0.4) |
| Control summary score | | | | | | | | |
| 6 months | 144 | 113 | 3.3  (2.7, 3.8) | 3.3  (2.8, 3.8) | 3.3  (2.7, 3.8) | 3.2  (2.8, 3.8) | 0.0  (-0.3, 0.5) | 0.0  (-0.5, 0.3) |
| 16-18 months | 100 | 90 | 3.2  (2.6, 3.8) | 3.2  (2.8, 3.8) | 3.3  (2.7, 3.9) | 3.2  (2.8, 4.0) | 0.0  (-0.5, 0.7) | 0.0  (-0.3, 0.5) |
| Support summary score | | | | | | | | |
| 6 months | 144 | 113 | 3.3  (2.8, 3.8) | 3.3  (2.7, 3.9) | 3.2  (2.6, 3.8) | 3.2  (2.7, 3.9) | -0.1  (-0.4, 0.3) | -0.1  (-0.4, 0.4) |
| 16-18 months | 100 | 90 | 3.4  (2.9, 4.0) | 3.3  (2.7, 4.0) | 3.2  (2.8, 3.9) | 3.3  (2.8, 3.9) | 0.0  (-0.4, 0.3) | 0.1  (-0.3, 0.4) |

^a^ Utrecht Work Engagement Scale, for each construct (vigour, dedication, absorption) responses are scored on a 7-point Likert scale, ranging from 0 ‘never’ to 6 ‘always (every day)’. Higher scores indicate greater work engagement.

^b^ Occupational Fatigue Exhaustion Recovery (OFER 15) Scale, for each subscale (chronic fatigue, acute fatigue, inter-shift recovery) responses are scored on a 7-point Likert scale, ranging from 0 ‘strongly disagree’ to 6 ‘strongly agree’. A score for each subscale is calculated which ranges from 0 to 100, with a higher score indicating a higher degree of the subscale construct.

^c^ Job satisfaction and performance were rated on 7-point Likert scales, ranging from 1 ‘dissatisfied / very poorly’ to 7 ‘extremely satisfied / extremely well’.

^d^ The total number of days participants reported being absent from work due to sickness over the last 6 months and the total number of days participants reported attending work despite not feeling well over the past six months.

^e^ Current workability rating, reported on a 11-point Likert scale ranging from 0 ‘worst’ to 10 ‘best’.

^f^ Perceived work demands, scored using a 5-point Likert scale ranging from 1 ‘never’ to 5 ‘always’. Higher calculated scores for each construct (demand, control, support) represent a higher degree of that construct.

**Table S12.** Markers of driving-related safety behaviour measured at baseline, at 6 months and at the final follow-up, along with changes calculated from baseline

| Variable | Number of participants | | Baseline  Median (IQR) | | Follow-up  Median (IQR) | | Median change from baseline to follow-up (IQR) | |
| --- | --- | --- | --- | --- | --- | --- | --- | --- |
|  | **Control** | **SHIFT** | **Control** | **SHIFT** | **Control** | **SHIFT** | **Control** | **SHIFT** |
| Occasionally jump to get out of lorry quickly | | | | | | |  |  |
| 6 months | 144 | 113 | 2  (1, 3) | 2  (1, 3) | 2  (1, 3) | 2  (1, 2) | 0  (-1, 0) | 0  (-1, 0) |
| 16-18 months | 100 | 89 | 2  (1, 3) | 2  (1, 3) | 1  (1, 3) | 1  (1, 2) | 0  (-1, 0) | 0  (-1, 0) |
| Compliance with posted speed limits | | | | | | | | |
| 6 months | 144 | 113 | 4  (4, 5) | 4  (4, 5) | 4  (3, 5) | 4  (4, 5) | 0  (-1, 0) | 0  (0, 0) |
| 16-18 months | 100 | 89 | 4  (4, 5) | 4  (4, 5) | 4  (4, 5) | 5  (4, 5) | 0  (0, 1) | 0  (0, 1) |
| Occasionally drive without getting enough sleep | | | | | | | | |
| 6 months | 144 | 113 | 3  (2, 4) | 3  (2, 4) | 3  (2, 4) | 3  (2, 4) | 0  (0, 0) | 0  (0, 1) |
| 16-18 months | 100 | 89 | 3  (2, 4) | 3  (2, 4) | 3  (2, 4) | 3  (2, 4) | 0  (-1, 0) | 0  (0, 1) |
| Always use logbook legally | | | | | | | | |
| 6 months | 144 | 113 | 5  (4, 5) | 5  (4, 5) | 5  (4, 5) | 5  (4, 5) | 0  (0, 0) | 0  (0, 0) |
| 16-18 months | 100 | 89 | 5  (4, 5) | 5  (4, 5) | 5  (4, 5) | 5  (4, 5) | 0  (0, 0) | 0  (0, 0) |
| Skip the daily vehicle inspection when tired or rushed | | | | | | | | |
| 6 months | 144 | 113 | 1  (1, 2) | 1  (1, 2) | 1  (1, 2) | 1  (1, 2) | 0  (0, 0) | 0  (0, 0) |
| 16-18 months | 100 | 89 | 1  (1, 2) | 1  (1, 2) | 1  (1, 1) | 1  (1, 1) | 0  (0, 0) | 0  (0, 0) |
| Sometimes get in a difficult situation without having a way out | | | | | | | | |
| 6 months | 144 | 113 | 2  (1, 3) | 2  (1, 3) | 2  (1, 3) | 2  (1, 2) | 0  (-1, 0) | 0  (0, 0) |
| 16-18 months | 100 | 89 | 2  (1, 3) | 2  (1, 3) | 2  (1, 3) | 1  (1, 2) | 0  (-1, 1) | 0  (-1, 0) |

Responses to each statement were scored using a 5-point Likert scale, ranging from 1 ‘strongly disagree’ to 5 ‘strongly agree’.
